# Overcoming the therapeutic resistance of hepatomas by targeting the tumor microenvironment

**DOI:** 10.3389/fonc.2022.988956

**Published:** 2022-11-15

**Authors:** Jiaxin Zhang, Huiqiong Han, Lei Wang, Wenjia Wang, Mei Yang, Yanru Qin

**Affiliations:** Department of Oncology, The First Affiliated Hospfigital of Zhengzhou University, Zhengzhou, China

**Keywords:** hepatocellular carcinoma, tumor microenvironment, therapeutic resistance, therapeutic targets, novel drugs, patient-derived organoids

## Abstract

Hepatocellular carcinoma (HCC) accounts for the majority of primary liver cancers and is the third leading cause of cancer-related mortality worldwide. Multifactorial drug resistance is regarded as the major cause of treatment failure in HCC. Accumulating evidence shows that the constituents of the tumor microenvironment (TME), including cancer-associated fibroblasts, tumor vasculature, immune cells, physical factors, cytokines, and exosomes may explain the therapeutic resistance mechanisms in HCC. In recent years, anti-angiogenic drugs and immune checkpoint inhibitors have shown satisfactory results in HCC patients. However, due to enhanced communication between the tumor and TME, the effect of heterogeneity of the microenvironment on therapeutic resistance is particularly complicated, which suggests a more challenging research direction. In addition, it has been reported that the three-dimensional (3D) organoid model derived from patient biopsies is more intuitive to fully understand the role of the TME in acquired resistance. Therefore, in this review, we have focused not only on the mechanisms and targets of therapeutic resistance related to the contents of the TME in HCC but also provide a comprehensive description of 3D models and how they contribute to the exploration of HCC therapies.

## Introduction

Primary liver cancer is one of the most aggressive and lethal cancers worldwide, with an increasing number of patients suffering from chronic liver fibrosis and inflammation (1). According to the Global Cancer Statistics 2020, hepatocellular carcinoma (HCC) ranks sixth in terms of cancer incidence and has a high mortality rate worldwide (2). A substantial proportion of primary liver cancers comprise HCC, intrahepatic cholangiocarcinoma, and other mixed tumors (3). HCC is the most common primary malignancy, is associated with a poor prognosis and recurrence within 5 years, and is often diagnosed at the end stage of the disease (4). Currently, liver resection and transplantation are the most promising curative options for patients with HCC. However, most patients diagnosed with HCC miss the optimal time window for surgery. Some palliative measures include locoregional therapies such as radiotherapy, trans-arterial chemoembolization (TACE), trans-arterial radioembolization (TARE), and ablation (microwave, cryoablation, and ethanol). In the past few years, there have been significant advances in chemotherapy, immunotherapy, and targeted therapies. However, drug resistance largely limits efficacy and has a negative impact on patient prognosis (5, 6). It is widely known that cancer therapeutic resistance mechanisms are complicated and are composed of two groups: intrinsic drug resistance (resistance factors that existed before drug treatment) and acquired drug resistance (caused by enhanced efflux of drugs, growth factors, increased metabolism of xenobiotics, enhanced DNA repair ability, and epigenetic factors among other factors during the treatment process) (7). Several studies have demonstrated that therapeutic resistance has a strong relationship with the tumor microenvironment (TME), mainly referred to as acquired resistance (8–10).

A prototype of the TME was initially proposed in 1889 as “seed and soil,” which vividly illustrated the relationship between tumor cells and the TME (11). This complex and dynamic TME mainly contains cellular components (cancer-associated fibroblasts [CAFs], immune cells, regulatory T cells [Tregs], myeloid-derived suppressor cells [MDSCs], tumor-associated macrophages [TAMs], and natural killer [NK] cells) and non-cellular components (tumor vasculature system, exosomes or extracellular vesicles (EVs), cytokines, and growth factors) (12, 13). Liver cancer is a rapidly growing solid tumor. The hypoxic microenvironment of liver cancer tissue is widespread, which not only stimulates the proliferation of liver cancer cells, causes angiogenesis, and accelerates invasion, but also has an important impact on drug tolerance (14). Moreover, dynamic changes in the TME mean that cells and extracellular secretions are constantly remodeled, which makes the microenvironment more conducive to the development of tumor drug resistance (15). Therefore, regulating the HCC microenvironment is an important treatment approach.

**Figure 1:**
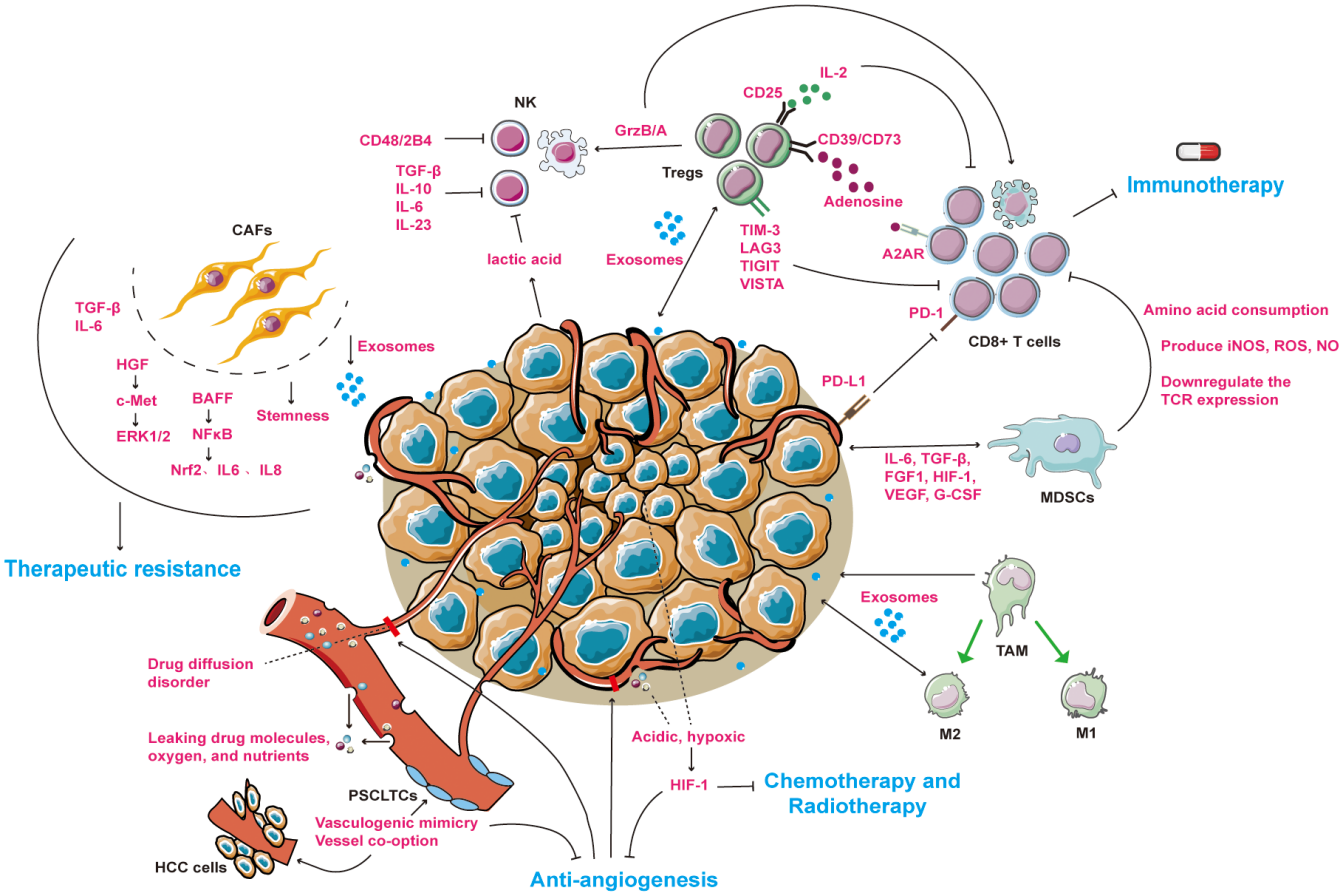
An overview of drug resistance mechanisms between tumor microenvironment and HCC cells. The microenvironment of HCC mainly includes cellular components (CAFs, NKs, Tregs, MDSCs, TAMs) and non-cellular components (tumor vasculature system, exosomes and cytokines). Under the combined effect of tumor and microenvironmental components, patients with liver cancer are resistant to immunotherapy, anti-angiogenesis or radiotherapy. → represents the pointing and promoting effect. ⊥ represents the inhibiting effect. The ↔ representative serves to enhance each other. The blue fonts represent the treatment for HCC patients. The red fonts represent microenvironmental molecules and their mechanisms of action. Black fonts represent cells name. CAFs, cancer associated fibroblasts; NKs, natural killer cells; Tregs, regulatory T cells; MDSCs, myeloid-derived suppressor cells; TAMs, tumor-associated macrophages; M1, tumor suppressor macrophages; M2, tumor-promoting macrophages. PSCLTCs, pluripotent stem cell-like tumor cells. HCC cells, hepatocellular carcinoma cells.

Immune checkpoint inhibitors (ICIs) and anti-angiogenesis therapies are the two main TME-targeted therapies; in particular, the IMbrave150 study demonstrated that the combination of the PD-L1 inhibitor atezolizumab with the anti-angiogenic agent bevacizumab had a survival benefit superior to the standard treatment of sorafenib in the order of many years (16). This regimen has also been approved by the FDA for the first-line treatment of HCC in many countries and regions. However, during treatment, new immune checkpoints, new angiogenesis patterns, and other factors in the microenvironment trigger acquired drug resistance and limit clinical efficacy. Therefore, targeting the TME as a battlefield to overcome many existing therapeutic limitations is a promising research direction. Simultaneously, it is necessary to accurately mimic the full appearance of the TME to explore tumor recalcitrant mechanisms and discover efficient therapies for HCC patients. For example, cultivation of three-dimensional (3D) organoid models *in vitro* provides a broader platform for preclinical studies. In this review, we mainly focus on the mechanisms and markers of drug resistance related to the HCC microenvironment (**Figure 1**) and elaborate on potential drugs for TME-targeted therapies. Furthermore, we discuss what the 3D organoid model is and how it contributes to HCC therapy exploration.

## Mechanisms of therapeutic resistance in the HCC microenvironment

### Vascular system

#### The emergence of vessel co-option and vasculogenic mimicry

Available data suggest that anti-angiogenesis can inhibit tumor growth, but compensatory angiogenesis is involved in anti-angiogenic therapeutic resistance. Important vascular stimulators have been studied in HCC, including vascular endothelial growth factor (VEGF)/vascular endothelial growth factor receptor (VEGFR), fibroblast growth factor (FGF)/fibroblast growth factor receptor (FGFR), platelet-derived growth factor (PDGF)/platelet-derived growth factor receptor (PDGFR), endoglin (CD105), and angioportin/tie (17). Among these factors, VEGF is the most important cytokine that facilitates angiogenesis and is composed of five different isoforms: VEGF-A, -B, -C, -D, and -E. The VEGF receptors VEGFR-1, -2, and -3 bind VEGF with different affinities (18). The combination of VEGF and VEGFR can not only trigger the proliferation of vascular endothelial cells but also activate lymphatic metastasis by forming new lymphatics. In 1971, Folkman suggested potential strategies against tumor progression by blocking tumor angiogenesis (19). Angiogenesis inhibitors are administered to patients with HCC, including bevacizumab, cabozantinib, lenvatinib, ramucirumab, regorafenib, and sorafenib, based on the National Comprehensive Cancer Network guidelines (20). Placental growth factor (PLGF) is a pro-angiogenic factor belonging to the VEGF family, which is usually secreted under pathological conditions. The overexpression of PLGF has been noted in several tumors resistant to anti-angiogenesis therapy, suggesting that PLGF is a prospective target in HCC treatment (21–24).

Although current anti-angiogenic drugs, together with immunotherapy, have prolonged the survival of patients to a certain extent, acquired resistance limits the therapeutic efficacy of HCC. To some extent, antiangiogenic drugs prevent the transport of chemotherapy drugs and limit the therapeutic efficacy. Furthermore, vasculogenic mimicry (VM) and vessel co-option (VC) are two emerging theories that explain resistance to anti-angiogenic drugs.

VM is a novel tumor microcirculation model independent of endothelial cells and was first developed by Maniotis et al. in 1999 (25). This model indicates that pluripotent stem cell-like tumor cells (PSCLTCs) acquire endothelium-like properties and form stable tubular structures without endothelial cells by secreting heparin sulfate, proteoglycans, collagen IV and VI, and laminin to transport nutrients for tumor growth (26). The VM process is unsurprisingly complex and is usually induced by a hypoxic TME and favored by many molecular mechanisms, mainly involving epithelial–mesenchymal transition (EMT), cancer stemness, response to hypoxia, and extracellular matrix remodeling (27, 28). VEGF/VEGFR inhibitors disrupt the formation of tumor blood vessels, leading to an acidic environment that compensates for the production of VM structures. Therefore, VM is an important cause of drug resistance after the application of anti-VEGF drugs to solid tumors. Sorafenib is a small-molecule tyrosine kinase inhibitor with anti-angiogenic activity. Clinical trials in breast cancer have been limited and have stopped at phase III. Mao et al. found that compared to the less invasive MCF7 cell line, only breast cancer stem-like cells (BCSLCs) and ALDH1+ MDA-MB-231 cells showed angiogenic ability and were directly involved in the development of VM. Sorafenib only inhibits endothelial angiogenesis and has no effect on VM. Mechanistically, they found that HIF-1α contributes to the proportion of BCSLCs (29). Furthermore, VM usually occurs in tumors with higher grades, shorter survival, and more aggressive disease (30, 31). Sorafenib resistance has been studied most frequently in HCC. Shi et al. found that the high expression of ITGA5 and ITGB1 in sorafenib-resistant liver cancer tissues promoted the degree of hypoxia and the generation of VM structures (32). The most important molecular mechanisms favoring VM in HCC include the following: (a) HIF1-α promotes VM production by regulating LOXL2 and is positively correlated with poor prognosis of HCC patients (33); (b) m6A methyltransferase METTL3 promotes VM generation and HCC metastasis by enhancing the translation efficiency of YAP1 mRNA and is related to Hippo pathway (34); (c) exosomes derived from hepatocytes, circRNA-100338, can affect the angiogenesis of liver cancer, including VM, thereby promoting the metastasis of liver cancer (35); (d) high expression of CD276 protein can activate the PI3K/AKT/MMPs pathway to promote VM formation, which is related with poor prognosis in HCC (36); (e) BMP4, migration-inducing gene 7 (MIG7), long noncoding RNA n339260, RhoC/ROCK2, Slug, and osteopontin were also found to be factors inducing VM formation in HCC (37–42); (f) EMT pathway can be induced by Hsp90B, Notch 1, ZEB2 and Twist1 to promote VM formation (43–46). These molecules are thought to be potential therapeutic targets for tumors resistant to anti-angiogenic therapy in patients with HCC.

Vessel co-option is a non-angiogenic mechanism in which tumor cells adhere to the existing normal vessels of host organs and grow infiltratively by migrating along them without angiogenesis (26). Kuczynski et al. performed an experiment to illustrate the co-option process in sorafenib-resistant HCC using Hep3B-hCG orthotopic HCC xenografts. Under sorafenib treatment, angiogenic vessels of the tumor are depleted but co-opted pre-existing vessels are preserved. Meanwhile, EMT-like molecules are increased, leading to both tumor invasion and incorporation into the liver parenchyma, co-opting the normal liver vascular system. These changes can be reversed by discontinuing sorafenib treatment (47, 48). Interestingly, following the onset of sorafenib resistance, tumor cells begin to surround the hepatic sinuses and major blood vessels and invade rapidly. The tumor invasion signal weakens and becomes angiogenesis-dependent only when sorafenib treatment is discontinued (49). In cases of colorectal cancer with liver metastases, vascular co-option is associated with resistance to anti-angiogenic therapy. Cancer cells replace hepatocytes by inducing apoptosis proteins, motility, and EMT, and enter sinusoidal vessels to establish vascular co-option (50). However, these findings lack specific markers of co-opted vessels and need to be verified in patients with HCC in future studies. If possible, anti-angiogenic drugs together with anti-co-opted vessels will be an optimal choice for patients with metastatic HCC.

#### Potential drugs and mechanisms to overcome antiangiogenic resistance in HCC

Recently, some VM-targeted drugs have been developed to inhibit VM in HCC and have shown satisfactory results in reversing drug resistance. The promising drugs and functional mechanisms that inhibit VM formation and overcome anti-angiogenic therapy resistance are summarized in **Table 1**.

**Table 1:** The novel targets and promising drugs to reverse resistance by inhibiting VM formation.

Molecule	Target	Target’s expression	Molecule function	Cell lines	Ref
Myricetin	PAR1	UP	Myricetin reversed PAR1-mediated EMT and inhibits HCC cell invasion, metastasis, VM formation and angiogenesis	PLC-PRF-5	(51)
Daurisoline	RhoA/ROCK2/AKT, ERK-p38 MAPK	UP	Daurisoline dramatically sensitized HCC cell lines to sorafenib	MHCC-97H	(52)
IU1 (S7134)	USP14	UP	IU1 treatment decreased cell proliferation, invasion, migration, and VM formation under hypoxia conditions	HCCLM3 Huh-7	(53)
Regorafenib	ID1, Snail VE-cadherin,	UP	Regorafenib distinctly inhibited EMT in HCC cells *via* targeting ID1, leading to the suppression of cell migration, invasion and VM formation.	Huh7, PLC/PRF/5	(54)
Androgen receptor (AR)	Notch4, VE-cadherin,	UP	AR suppressed the VM formation by down-regulating circRNA7/miRNA7-5p/VE-Cadherin/Notch4 signal	SKhep1, HA22T	(55)
Y27632	ROCK	UP	Y27632 inhibited VM formation *via* TGF-B1/ROCK induced EMT pathway	HepG2, MHCC-97H	(56)
Melittin	HIF-1*α*, p-AKT, MMP2/9	UP	Melittin suppressed VM formation by inhibiting HIF-1*α*/AKT pathway	SMMC-7721 Huh7, HepG2	(57)
NVP-BEP800	Hsp90B Twist1	UP	NVP-BEP800 suppressed VM formation by releasing Hsp90B and Twist1 interaction.	SMMC-7721	(43)
Biejiajian Pills	VE-cadherin, PI3K, RhoA ROCK	UP	Biejiajian Pills can inhibit the formation of VM in HCC cells *in vitro* possibly by inhibiting the RhoA/ROCK pathways and the expressions of VE-cadherin and PI3K.	HepG2	(58)
Polyphyllin I (PPI)	Twist1 VE-cadherin	UP	PPI impaired VM formation by decreasing Twist1 and VE-cadherin, and blocking PI3k/Akt pathway	SMMC-7721 PLC/PRF/5	(59)
Arsenic trioxide (As2O3)	VE-cadherin, MMP2, MMP9	UP	As2O3 inhibited VM formation through downregulating the expression of VE‐ cadherin, MMP2, and MMP9	HepG2	(60)

### Immune system

#### The immunosuppressive environment contributes to therapeutic resistance

The imbalance between the recruitment of immunosuppressive cells (MDSCs, TAMs, and Tregs) and the reduction of anti-tumor effector cells (cytotoxic T lymphocytes [CTLs], NKs, and dendritic cells [DCs]) results in an immunosuppressive microenvironment for HCC, leading to immunotherapy resistance (61). A high-level overview of these mechanisms is shown in **Figure 1**. The mechanisms and potential targets of drug resistance in these cells are described.

Tregs are a subset of CD4+CD25+ immunosuppressive T cells, characterized by Forkhead box protein P3 (Foxp3) expression, which determines the development and differentiation of Tregs. In HCC tissues, a high concentration of Tregs combined with a low concentration of CD8+T cells is an independent factor that affects the survival and recurrence of patients (62). However, the mechanisms by which Tregs induce resistance to ICIs and sorafenib in HCC have not been fully elucidated. However, a number of molecules and signaling pathways have been implicated in Treg-induced immune tolerance: (a) the emergence of other immune checkpoints from Tregs, such as TIM-3, TIGIT, LAG3, and VISTA, blocks effector T cell activation, and their high expression is often associated with poor prognosis in HCC patients after anti-PD-1/PD-L1 treatment (63, 64); (b) TGF-B secreted by Tregs induces EMT and promotes the invasion and migration of HEPA1-6 cells. The combination of TGF-B inhibitors significantly enhances the sensitivity of HCC cells to regorafenib and sorafenib (65, 66); (c) CD25 expressed by Tregs competitively consumes IL-2, reduces effector T cell activation, and induces metabolic disorders (67); (d) CD73, CD39, and cyclic AMP-regulated adenosine A2 receptor (A2AR) can reduce T-cell toxicity and shift the microenvironment into a tolerant state (68); (e) Tregs secrete granzyme B/perforin that directly lyses NK and CD8+ T cells (69). Moreover, CCR4+ Tregs have been found to be an important type of Tregs in HBV^+^ HCC, correlated with sorafenib resistance and increased IL-10 and IL-35 levels, and treatment with a CCR4^+^ antagonist has been shown to successfully reverse sorafenib resistance and sensitize liver cancers to PD-L1 checkpoint blockade (70).

MDSCs are heterogeneous and immature cells derived from the bone marrow, which can be induced in almost all types of tumors and in pathological conditions such as infection, autoimmune disease, and trauma. The mechanism of drug resistance in MDSCs is mainly through damaging T cell function: (a) MDSCs overconsume essential amino acids such as arginine-1 and cysteine in the body, leading to T cell proliferation and activation dysfunction, as well as tryptophan by overexpression of indoleamine-pyrrole 2,3-dioxygenase (IDO) (71–73); (b) Cystine deprivation downregulates the T-cell receptor (TCR) ζ chain of hepatic CD8(+) T cells and helps tumor cells to escape; and (c) MDSCs also express cytotoxic reactive oxygen species (ROS), inducible nitric oxide synthase (iNOS), and nitric oxide (NO) to influence T cell viability (74). Furthermore, many tumor-derived cytokines such as FGF1, HIF-1, VEGF, IL-6, and G-CSF have been shown to promote the accumulation of MDSCs and are related to therapy resistance (75–80). For example, Xu et al. argued that chemotherapy-resistant HCC cell-released IL-6 boosts the silencing and activity of MDSCs and, when anti-IL6 neutralizing antibody is combined with 5-FU chemotherapy, tumor growth is significantly decreased (80). TGF-B secreted by MDSCs participates in a variety of drug resistance mechanisms, such as EMT induction, CSC promotion, and immunosuppression (81). Downregulation of TGF-B expression can overcome sorafenib resistance in liver cancer (82). Furthermore, hepatoma-intrinsic CCRK inhibition reduces MDSCs immunosuppression and enhances the blocking effect of the immune checkpoint (PD-L1) (83). SB265610, a CXCR2 antagonist, may reverse immunosuppression by inhibiting the chemotaxis of MDSC to the tumor, thereby promoting the anti-tumor immunity of CD8+ T cells and inhibiting tumor immune escape (84). MDSC-targeted therapy has achieved great progress in solid cancers, such as melanoma, breast cancer, NSCLC, and glioblastoma, and has even entered the stage of clinical trials, but research on liver cancer is still lacking.

TAMs are another type of marrow-derived cells that are divided into M1 (tumor suppressor macrophages) and M2 (tumor-promoting macrophages) subtypes, with type M2 predominating in liver cancer (85). In multiple cancers, the infiltration of M2 TAMs is highly related to poor prognosis and treatment resistance (86, 87). The main mechanism by which TAMs exert immune tolerance in HCC is through the secretion of molecules. For example, hepatocyte growth factor (HGF), derived from polarized M2 TAMs, confers HCC resistance to sorafenib in a feed-forward manner. Accumulated HGF can activate the HGF/c-Met, ERK1/2/MAPK, and PI3K/AKT pathways in tumor cells, recruit more M2 TAMs, and promote tumor growth. Therefore, the combination of the HGF inhibitor cabozantinib and sorafenib is reasonable for improving the efficacy of first-line systemic therapies (88). TAMs can also induce oxaliplatin resistance through autophagy in HCC cells (89). Therefore, blocking the recruitment of TAMs or reprogramming the polarization of TAMs is a promising measure to enhance the sensitivity of HCC treatment. Antagonists targeting the chemokine C-C motif ligand 2 receptor (CCR2) and stromal cell-derived factor 1 α (SDF-1α/CXCL12) have shown good efficacy in blocking TAM recruitment (90–92). Targeted colony stimulating factor (CSF-1), autophagy, NF-KB, Mir-214, IL-6, and toll-like receptors (TLRs) can transform TAMs from a pro-tumor phenotype into an antitumor phenotype (M1) (93–99).

NK cells are abundant in liver tissue, exerting their ‘killing’ function by regulating the activation receptors (NKG2C, NKG2D, CD244, CD266, and NCR) and inhibitory receptors (CD94/NKG2A and KIR) expressed on the surface of NK cells (100). The balance between the signals elicited by these receptors determines whether NK cells are activated and perform their effector functions. NK cell abnormalities induced by the TME are the main reason why tumor cells escape the immune response. For example: (a) immunosuppressive factors such as TGF-B, IL-10, IL-6, and IL-23 secreted by Tregs, MDSCs, or TAMs suppress NK cell function and induce tumor evasion and progression (101); (b) NK cells are excessively activated by mononuclear macrophages through CD48/2B4 interactions, thus inducing NK cell exhaustion and death (102); and (c) increased lactic acid content in tumor cells leads to metabolic reprogramming, resulting in NK cell dysfunction (103). In addition, the influence of HCC cells and their microenvironment can limit the sensitivity to NK cell cytotoxicity. NK cells targeting CSCs are known to inhibit tumor progression; however, the resulting overexpression of CEACAM1 renders EpCAM^+^ HCC cells resistant to NK toxicity. Anti-CEACAM1 antibody has been shown to restore the cytotoxicity of NK cells against EpCAM^+^ Huh-7 cells (104). Similarly, the inhibition of CNOT7 and the enhancement of zeste homolog 2 (EZH2), miR-889, granulin-epithelin precursor (GEP), and MICA/B can successfully reverse NK cell resistance, suggesting that these molecules are promising targets for immunotherapy in HCC patients (105–109).

#### Targeting immune environment to overcome drug resistance in HCC

As mentioned above, the immune system greatly influences tumor response to various treatments in the microenvironment. Therefore, based on the above immune tolerance mechanisms, significant efforts have been made to attempt to reverse tumor drug resistance, including: (a) combining immune checkpoint inhibitors; (b) inhibiting the recruitment of Tregs, MDSCs, and M2 TAMs in tumor tissue; (c) reprogramming the polarization of TAMs; and (d) reinforcing the anti-tumor capabilities of the immune system.

Multiple studies have shown that PD-1/PD-L1 inhibitors in combination with multi-tyrosine kinase inhibitors, VEGF inhibitors, or CTLA-4 inhibitors are superior to monotherapy (110). Two CTLA-4 inhibitors, ipilimumab and tremelimumab, have recently gained increasing attention, with FDA approval in 2011 for patients diagnosed with advanced or unresectable melanoma and in 2015 for patients with malignant mesothelioma (111, 112). In contrast to the immunonormalizing effect of PD-1/PD-L1 inhibitors, CTLA-4 suppression tends to largely enhance the toxic function of T cells, thus increasing the sensitivity to other treatments. Currently, the combination of ipilimumab and nivolumab/pembrolizumab has been associated with encouraging survival outcomes in patients with advanced HCC with primary resistance to prior immune checkpoint inhibitors (113). Moreover, this combination as a first-line therapy for patients with advanced HCC is currently undergoing evaluation (NCT04039607). Tremelimumab, another CTLA-4 inhibitor, in combination with durvalumab for patients with unresectable hepatocellular carcinoma, has been associated with a reasonable outcome (median overall survival of 18.7 months) and a satisfactory benefit-risk profile (114). With the emergence of more immune checkpoints and anti-PD-1/L1 resistance, the combination of immune checkpoint inhibitors to overcome drug resistance has become a popular trend. For example, the inhibition of TIGIT together with anti-PD-1 has been shown to significantly decrease tumor growth and increase the proportion of cytotoxic T cells in tumors (64). Fibrinogen-like protein 1 (FGL1) was recently identified as a major immune inhibitory ligand of LAG-3 and its overexpression was positively associated with poor prognosis, anti-PD-1 therapy resistance, and sorafenib resistance (115, 116). Although the exact mechanism by which FGL1/LAG3 regulates the immune environment remains unclear, synergistic inhibitory effects of anti-FGL1 and anti-PD-1 have been identified in animal studies. Similarly, a combined inhibitory effect of anti-LAG-3 and anti-PD-1 has been identified in several cancers such as melanoma, NSCLC, and breast cancer (117–119). Therefore, FGL1 and LAG3 can be used as next-generation immunotherapy targets independent of PD-1/PD-L1.

Anti-tumor efficacy can be enhanced by inhibiting Tregs, MDSCs, and TAMs, or by targeting important molecules to enhance NK cell activity. Related targets and potential reversal of drug resistance are described in **Section 2.2.1**. Adoptive cell transfer is an emerging therapeutic strategy with wide potential value in the treatment of HCC, and is deemed to be a highly individualized cancer therapy because of the adhibition of the patients’ own effect factors (120). What’s more, antiviral treatment for HBV-HCC was proved effective in increasing the postoperative survival (121). In the clinical study of PD-1 antibody, when ICIs repair immune function, HBV infection can be significantly reduced. In mouse models, it can also be seen that the virus infection rate of mice treated with PD-1 antibody is significantly reduced. Thus, the immune escape mechanisms of viruses and tumors actually work in the same pathway (122). Therefore, it is worth exploring whether there is a relationship between viral load and treatment resistance and whether the combination of antiviral drugs and immunotherapy or anti-angiogenesis is more effective. At present, adoptive cell therapy has been developed into a phase III clinical treatment, including chimeric antigen receptor (CAR) T cell therapy, tumor-infiltrating lymphocyte (TIL) therapy, engineered TCR therapy, cytokine-induced killer (CIK) cell therapy, and NK cell therapy. However, more effort is needed to achieve the desired clinical effect. Thus, due to the high heterogeneity of HCC tissues, targeting the immune system requires a combination of other molecular targeting inhibitors or multiple types of therapies.

### Cancer-associated fibroblasts and therapeutic resistance

It has been well documented that CAFs are the paramount population of stromal cells responsible for modulating neighboring cancer cells by way of autocrine, paracrine, and exosome functions (123). CAFs and their secreted soluble factors including cytokines (TGF-B, IL-4, IL-6), chemokines (CCLX and CXCL family members), pro-angiogenesis factors (VEGF, PDGF, and HIF), enzymes (MMPs), and ECM proteins (ectodysplasin-A and collagen type-I) contribute to tumor progression (124, 125). CAFs are highly heterogeneous and lack clear markers to distinguish functional subsets, which usually induces worse outcomes in anticancer therapy. With the participation of TME, CAFs are found contributory to increase the chemoresistance of HCC from multiple mechanisms (126–128).

First, paracrine signaling plays an important role in crosstalk between CAFs and tumor cells. For example, CAF-derived HGF increases the resistance of CD73^+^ cancer cells to sorafenib or cisplatin through the HGF-c-Met-ERK_1/2_ pathway in HCC (129). CAF-derived TGF-B, as the most crucial characteristic in the HCC inflammatory process, is becoming an inducer of resistance in various tumors, including HCC (126, 130–132). Recently, Liu suggested that valproic acid (VPA) could reverse TGF-B-induced sorafenib resistance in HCC cells by reducing the Jagged2-mediated Notch1 signaling pathway and altering the EMT phenotype (133). CAF-derived IL-6 can impair the activity of tumor-infiltrating T cells and neutralization of IL-6 reverses anti-PD-L1 resistance in an HCC mouse model (134).

On the contrary, cancer cells affect the activation and expression of CAFs to protect themselves from toxic drug attacks. Gao et al. noted that when co-cultured with sorafenib-resistant Huh7 cells, the BAFF/NF-KB axis could be activated in CAFs and simultaneously induce the upregulation of Nrf2, IL6, and IL8, which contributed to the development of drug resistance in non-resistant Huh7 cells (135). The positive feedback of the CAF-HCC cell loop makes the tumor more resistant.

Furthermore, in view of the critical role of CSCs in therapy resistance, CAFs can promote stem cell action to increase cancer cell resistance. The C-MET/ERK/FRA1/HEY1 axis is mediated by CAF-derived HGF to promote the stemness of tumor-initiating cells (136). In line with HGF, IL-6 can regulate STAT3/NOTCH1/NICD/HES1 signaling to enhance the stem cell-like properties of HCC, either (137). The neutralizing antibody HGF or IL-6 has been used to successfully reverse the stem cell viability of CD24+ HCC cells *in vitro* and *in vivo* through the HGF/C-MET/STAT3 or IL6/IL6R/STAT3 signaling pathways (138). COMP, derived from CAFs, can induce EMT and stemness in HCC cells. RvD1 has been shown to damage COMP by targeting FPR2/ROS/FOXM1 signaling to eliminate the recruitment of COMP promoters by FOXM1 (139). The cellular crosstalk between CAFs and HCC cells does not stop. CAF-derived CLCF1 increases the self-renewal ability of HCC cells by binding to CNTFR expressed in an autocrine manner. Cancer cells then secrete CXCL6 and TGF-B, which partially account for the CLCF1-regulated stemness of HCC cells (140). Moreover, cessation of autophagy is also an effective way to attenuate CAF-promoted stemness in HCC (141).

Finally, some small molecules carried by CAF-derived exosomes help markedly. CAF-derived exosomes containing circZFR enhance chemoresistance to cisplatin by inhibiting the STAT3/NF-KB pathway in HCC (142). CAF-secreted exosomal miR-1247-3p is associated with the production of IL-6 and IL-8 in CAFs through B1-integrin–NF-KB signaling, which increases the stemness and EMT of HCC (143). Evidence suggests that DNA methyltransferase 3 beta (DNMT3b) is upregulated in HCC tissues and is associated with poor progression. DNMT3b-targeted therapy with annamycin A has been shown to significantly reverse sorafenib resistance in cells (144). Moreover, CAF-derived exosomal miR-29b can inhibit HCC progression by targeting DNMT3b, serving as a potential drug (145).

In conclusion, the above literature findings illustrate the mechanisms of drug resistance between CAFs and HCC cells, suggesting that CAF-targeted therapy is a promising method for reversing drug resistance in HCC (**Table 2**). Apart from the inhibitors mentioned above, some traditional Chinese medicines that exert antifibrotic properties have been reviewed recently (146). Several anti-HGF/c-MET antibodies, such as onartuzumab, tepotinib, capmatinib, and tivantinib are already in the clinical stage of HCC treatment evaluation (NCT01897038, NCT02115373, NCT01737827, NCT02029157).

**Table 2:** CAFs-derived molecules promote chemotherapy resistance in HCC.

CAFs-derived molecules in HCC	Effect on HCC	Mechanism	Referance
HGF	Resistance to sorafenib or cisplatin	HGF enhances the resistance of CD73^+^ cancer cells to sorafenib or cisplatin through HGF-c-Met-ERK_1/2_ pathway	(129)
	Induce stemness	C-MET/ERK/FRA1/HEY1 axis is mediated by HGF to promote the stemness of tumor-initiating cells	(136)
TGF-B	Resistance to sorafenib	TGF-B induces stemness, EMT and metabolic reprogramming in CAFs	(126)
IL-6	Resistance to anti-PD-L1 therapy	Activated IL-6/STAT3 signaling can resist anti-PD-L1 therapy in HCC	(99)
COMP	Induce EMT and stemness	COMP is derived from CAFs in a paracrine manner and initiate EMT and resistance in HCC.	(139)
CLCF1	Induce stemness	–	(140)
CircZFR	Resistance to cisplatin	Exosomal circZFR enhanced chemoresistance to cisplatin by inhibiting the STAT3/NF-KB pathway of HCC cells	(142)
MiR-1247-3p	Induce EMT and stemness	Exosomal miR-1247-3p was associated with the production of IL-6 and IL-8 in CAFs through B1-integrin–NF-KB signaling	(143)

### Hypoxia

#### Hypoxia and acidic environment

Abnormal vascular morphology in HCC tumors results in a preliminary anoxic and acidic microenvironment after the treatment of anti-angiogenic therapy (147). The lack of oxygen and nutrients often causes cancer cells to undergo glycolysis rather than metabolism, producing large amounts of lactic acid. The degree of hypoxia in the HCC microenvironment is heterogeneous. As the degree of hypoxia increases along with depth from the blood vessels into the tumor, HCC cells are exposed to a smaller concentration of oxygen and the pH decreases (148, 149). Tyrosine kinase inhibitors, anti-angiogenic drugs, or other treatments that restrict the blood supply within the tumor can exacerbate the development of an anoxic and acidic environment, leading to tumor progression and drug resistance (150). Hypoxia-regulated transcription factor HIF-1 is an extensively authoritative marker to modify cancer sensitivity to therapeutic agents, and comprises a heterodimeric DNA-binding complex composed of α and B subunits (151–154). Hypoxia-induced HIF-1α elevation and multiple mechanisms contributing to chemotherapy resistance are related to: (a) alterations in metabolic pathways. Glucose uptake and glycolysis are strongly activated in hypoxic environments to meet the demands of tumor cell growth. HIF-1α induces many glycolytic genes including glucose transporter type 1 (GLUT1) and hexokinase 2 (HK2) (155, 156). Genistein, as a natural isoflavone, can directly downregulate HIF-1α, thus inactivating GLUT1 and HK2 to inhibit aerobic glycolysis, and therefore induce apoptosis of aerobic glycolysis HCC cells. At the same time, Genistein enhanced the sensitivity of sorafenib resistant HCC cells *in vivo* and *in vitro* (157); (b) crosstalk between autophagy and mitophagy. In the context of increased metabolic demands, mitophagy and autophagy may play key roles in inducing chemoresistance by regulating the adaptation of tumor cells to hypoxia, increasing oxidative stress and DNA damage, and providing nutrition and energy to tumor cells (158). B-cell lymphoma-2/adenovirus E1B 19 kDa-interacting protein 3 (BNIP3) and BNIP3-like protein X (NIX) are hypoxia-induced HIF-1α target genes that can bind LC3 and trigger the mitophagy response (159). Upregulation of BNIP3 by HIF-1α can promote autophagy and oxidative resistance in HCC cells (160). However, treatment with sorafenib did not inhibit its cytoprotective action (161); (c) drug efflux. ATP-binding cassette (ABC) transporters, such as MDR1, can pump chemotherapeutic drugs from the intracellular to extracellular regions of HCC cells to produce drug resistance. The reporter gene assay and electrophoretic mobility shift assay proved that HIF-1α is a critical factor for MDR1 gene overexpression (162); and (d) apoptosis inhibition. HIF-1α is the promoter activator of CBR1, and CBR1 overexpression induces apoptosis resistance by reducing oxidative stress associated with hypoxia, cisplatin, and doxorubicin treatment (163). Furthermore, several important signaling pathways and other gene responsible for chemotherapy resistance under hypoxia are summarized in **Table 3**; however, whether they are regulated by HIFs has not been verified.

**Table 3:** hypoxia related gene targets and mechanisms induced chemotherapy resistance in HCC.

Mechanism Type	Targets	HIF-1αrelated	Mechanism	Reference
Mitophagy	ATAD3A	Unknown	MiR-210-5P/ATAD3A/PINK1/PARKIN axis regulates hyperactivated mitophagy to induce sorafenib resistance in HCC under hypoxia.	(164)
	NIX and BNIP3	Yes	NIX and BNIP3 are HIF-1α mitotic targets related to mitophagy activity to induce sorafenib resistance.	(161)
Autophagy	FOXO3	Unknown	RNA m6A methylation regulates sorafenib resistance in liver cancer through FOXO3-mediated autophagy in hypoxic TME.	(165)
	FOXO3a	Unknown	FOXO3a-dependent transcriptive activation of beclin-1 is responsible for hypoxia-induced autophagy in sorafenib-resistant HCC	(166)
	BAG5	Unknown	Deletion of PRMT6 induces autophagy and promotes drug resistance of HCC by regulating the stability of BAG5-related HSC70 through post-translational methylation of BAG5	(167)
	14-3-3ζ/beclin1	Unknown	14-3-3ζ binds to and stabilizes phospho-beclin-1(S295) and induces autophagy in HCC cells to resist cis-diammined dichloridoplatium in hypoxic TME.	(168)
	ADRB2	Yes	ADRB2 signaling negatively regulated autophagy, leading to hypoxia-inducible factor-1α stabilization, reprogramming of hepatocellular carcinoma cells glucose metabolism, and the acquisition of resistance to sorafenib.	(169)
	Egr-1	Unknown	Hypoxia-induced Egr-1 expression enhanced drug resistance of HCC cells likely through autophagy.	(170)
	BNIP3	Yes	Upregulation of BNIP3 contributes to autophagy and anoikis resistance of HCC cells	(160)
Apoptosis inhibition	carbonyl reductase1 (CBR1)	Yes	Hif-1α can activate the promoter of CBR1, and CBR1 overexpression can inhibit apoptosis by reducing oxidative stress under hypoxia, cisplatin and doxorubicin treatment.	(163)
	COX-2	Yes	COX 2 induces apoptosis resistance *via* HIF 1α/PKM2 pathway in HCC cells.	(171)
	YAP and TAZ	Unknown	YAP and its paralog TAZ induce apoptosis resistance of HCC cells under hypoxia.	(172)
Drug efflux	ABCB1	Unknown	NRF2/ABCB1-mediated efflux and PARP1-mediated DNA repair contribute to doxorubicin resistance in chronic hypoxic HepG2 cells.	(173)
	ABCG2	Yes	ABCG2-mediated drug efflux induces cancer stemness of HCC cells	(174)
Metabolic pathways	GLUT1/3	Yes	GLUT1 and GLUT3 are upregulated to enhance the resistance to 5-caffeylquinic acid	(175)
	USP29	Yes	USP29 promotes sorafenib resistance by upregulating glycolysis	(176)
	MCT-4	Yes	Glycolysis conversion of HIF-1 and McT-4 reduces hepatocellular carcinoma cell apoptosis	(177)
	HK2	Yes	Hypoxia prevents hepatocellular carcinoma cell apoptosis through HIF-1α-dependent induction of hexokinase II expression	(155)
Important signaling pathways	NF-KB axis	Yes	NF-KB activation induce HMGB1 mediated cisplatin resistance or sorafenib resistance.	(178, 179)
	ERK/MAPK axis	Yes	ERK/MAPK pathway promotes the formation of MDR through P-gp, MRP1, LRP genes	(180)
	NPM1/PTPN14/YAP axis	Unknown	NPM1/PTPN14/YAP axis mediates the hypoxia-induced chemoresistance to sorafenib	(181)
	mTORC1/ p70S6K/RP-S6 axis	Yes	mTORC1/p70S6K/RP-S6 axis is a target to reverse the resistance to sorafenib by preventing HIF-1α synthesis to block the cytoprotective mitophagy induced by the hypoxic microenvironment.	(161)
Other	FBI-1	Yes	FBI-1 regulates the miR-30c/HIF-1α to promote the Warburg effect and enhance the resistance to molecular targeted agents	(154)

Hypoxia is a typical factor that leads to radiotherapy resistance in solid tumors. Radiation can directly damage the DNA strands or damage the DNA strands through the production of radicals, resulting in the death of tumor cells (182). In an aerobic environment, oxygen reacts with free radicals on broken DNA strands, forming a more stable pattern of DNA damage. It’s also a stable hydrogen peroxide structure, which can promote more DNA breaks. In anoxic environments, tumor cells repair DNA damage by removing hydrogen from free sulfhydryl groups, thereby developing resistance to radiotherapy (183). Similarly, HIF-1α overexpression during hypoxia is an important factor in radioresistance. Bai Bing et al. found that inhibition of the EZH2/Mir-138-5p/HIF-1α pathway could enhance the sensitivity of radiotherapy (184). In addition, the activation of PI3K/AKT signaling is known to induce radioresistance in various tumors by increasing HIF-1α translation efficiency, and elevated PDK1 is a driver of PI3K/AKT/mTOR signaling in HCC, suggesting that this pathway is a potential therapeutic target to reverse radiotherapy resistance (185, 186). Furthermore, HIF-1α can also be upregulated by mTORC1 to confer radiotherapy resistance to HCC through the anabolic integration of glucose and cardiolipin (187).

#### Targeting anoxic and acidic environment to reverse drug resistance

Hypoxia is a characteristic of HCC and contributes to chemotherapy and radiotherapy resistance. Therefore, reprogramming the hypoxic environment using immunosuppressants, nanoparticles, natural products, and HIF-1α inhibitors is an attractive direction for future research. Rapamycin, an mTOR inhibitor, reverses resistance to adriamycin during hypoxia by decreasing hypoxia-induced HIF-1α accumulation (188). Moreover, rapamycin can increase the sensitivity of HCC cells to cabozantinib (a c-Met inhibitor), which has a synergistic inhibitory effect on HCC cells (189). Nanoparticles are an emerging tool to improve the hypoxic environment of HCC. Nanoparticle delivery of oxygen-generating MnO_2_ and anti-angiogenic drugs can normalize the TME by inhibiting hypoxia-induced invasion, EMT, and metastasis, and increasing the expression of M1-type macrophage genes (Nos2, IL-6, CCL5, CXCL9, and CXCL11) (190). Bruceine D and silymarin are two newly discovered natural products that reverse drug resistance by decreasing HIF-1α expression and can directly block the inhibitor of B-catenin and T-cell factor/B-catenin interaction to regulate HCC cell metabolism, and silymarin reduces the expression of MDR1 and P-glycoprotein (191, 192). Furthermore, sorafenib inhibits HIF-1α synthesis and shifts the hypoxia response from the HIF-1α- to HIF-2α-dependent pathway. HIF-2α overexpression activates the TGF-α/EGFR pathway, thereby inducing sorafenib drug resistance. This means that to inhibit cancer growth, it may be necessary to target both HIF-1α and HIF-2α. The HIF-1α mRNA antagonist RO7070179 has shown primary success in a phase Ib study (NCT02564614).

### Exosomes

#### TME-derived exosomes and therapeutic resistance

Exosomes function as vectors to transport a large number of molecules between cells in the TME, including messenger RNA (mRNA), long noncoding RNA (lncRNAs), microRNAs (miRNAs), circular RNA (circRNAs), lipids, proteins, and nucleic acids. These molecules are secreted by cancer or stromal cells in the TME and are involved in TME remodeling, tumor progression, invasion, angiogenesis, metastasis, and drug resistance (193). On one hand, exosome-associated therapeutic resistance is achieved through transport from drug-resistant cancer cells to drug-sensitive cells. For example, Fu et al. found that exosome miR-32-5p from a multidrug-resistant cell line (Bel/5-FU) activated the PI3K/Akt signaling pathway by inhibiting PTEN and induced drug resistance in sensitive cells by facilitating EMT and angiogenesis (194). MiR-221/222 from chemotherapy-resistant MCF-7 cells can also render sensitive MCF-7 cells resistant, but this has not been verified in HCC (195). In contrast, exosomes may swallow drug molecules and pump them out with the help of the ATP-binding cassette (ABC) transporter family (such as ABCB1) (196, 197). Recently, several researchers have made progress in the study of exosome drug resistance in HCC. Li et al. found that miR-27a-3p derived from M2 macrophage exosomes is responsible for HCC stemness and directly negatively regulates TXNIP, which has been reported as a tumor repressor gene. Upregulation of TXNIP can reverse drug resistance induced by elevated miR-27a-3p levels (198). Zhang et al. found that overexpressed plasma exosome circUHRF1 could increase TIM-3 expression caused by miR-449c-5p decline to inhibit NK cell function and induce anti-PD-1 therapy resistance in patients with HCC (199).

#### Targeting TME-derived exosomes in HCC has advantages and disadvantages

Communication between cells is a double-edged sword that can not only transmit information to each other to promote the occurrence and development of tumors and drug resistance but also provide treatment targets. In fact, some exosome-mediated RNAs have shown satisfactory potential in relieving therapy-resistant stress. MiR-199a-3p, miR-744, and miR-122 are downregulated in HCC tissues, and chemotherapy resistance in HCC has been successfully reversed through exosome transport (200–202). Furthermore, exosomes as drug carriers have great advantages and a good precedent for the treatment of HCC patients. First, compared to artificial liposomes, exosomes have higher packaging efficiency and stability. Second, exosomes can reduce immune responses *in vivo*. Third, because of the specific molecules on the exosome surface, interaction with antibodies or coagulation factors is limited, thereby reducing the occurrence of immune responses *in vivo* (203). Notably, several major applications in exosome packaging include small-molecule chemical drugs, proteins, peptides, and nucleic acid drugs. Zhang et al. designed a neutrophil-derived exosome-like vesicle loaded with doxorubicin and decorated it with superparamagnetic iron oxide nanoparticles, which showed precise targeting in gastric cancer, hepatoma, and colon cancer cell lines (204). Another exosomal miR-155 inhibitor markedly improved cisplatin susceptibility in a cisplatin-resistant oral squamous cell carcinoma 3D model by decreasing MET and drug efflux transporter proteins (205). Although great progress has been made over the last few decades and multiple preclinical trials show promising results, there are still obstacles to be overcome (206). For example, there is a lack of standardized exosome isolation and purification techniques. Traditional separation techniques are often contaminated by other types of exosomes, which can significantly affect therapeutic efficiency. Second, thorough and accurate research on exosome characterization is required because exosomes from different cell sources may have opposite therapeutic effects. These technical barriers must be addressed so that exosomes can be used for the diagnosis or prognostic monitoring of cancer and innovative, personalized exosome-based therapies.

## Organoid models for better understanding therapeutic resistance in HCC

An organoid is a type of three-dimensional micro-organ cultivated *in vitro*, which has a complex structure similar to real organs and can partially simulate the physiological function of the source tissue or organ. In early 2009, Clevers et al. constructed the first mouse gut organoids with single sorted Lgr5(+) stem cells, which maintain the ability of self-renewal and maintain the villous structure of the intestinal gland fossa (207). Over the past five years, research on human-derived organoids in preclinical platforms has developed rapidly. Compared with two-dimensional (2D) cell line cultures, organoids can mimic tumor heterogeneity, cell-cell interactions, and cell-extracellular matrix communication, although not completely (208). Compared to the disease mouse model, organoids have physiological and pathological characteristics that are unique to humans (209). Based on these advantages, organoids can provide more possibilities for personalized therapy, not only to verify the mechanism of the microenvironment in tumors *in vitro*, but also to facilitate the progress of new drug screening. In addition, the fact that many drugs show positive results *in vitro* or in mouse models followed by negative effects in clinical trials underscores the importance of exploring realistic hepatocellular organoid models to simulate efficacy.

### Maturation of human hepatocyte organoids

Since the first attempt to construct a 3D cell culture system in the 1990s, the development of human hepatocellular organoids has gradually matured (210). Organoid material sources range from mice to humans, and the maintenance of organoid function increases from a few days to eight months. The research scope of organoids in patients with liver cancer has gradually expanded, including exploration of the mechanism of microenvironmental drug resistance, drug screening, and personalized treatment. **Table 4** shows several typical examples of the maturation process of hepatocellular organoid development.

**Table 4:** The maturation process of hepatocellular organoid development.

Cellular source	Characteristics	Meaning	Reference
Single mouse Lgr5+ liver stem cells	Such clonal organoids can be induced to differentiate *in vitro* and to generate functional hepatocytes upon transplantation into Fah(-/-) mice.	Mark the first organoid from murine	(211)
Adult hepatic duct cells	Clonal organoids are genetically stable	Mark the first organoid from human	(212)
Commercially purchased HCC cells	Initiating the co-culture with non-parenchymal cells such as fibroblast and endothelial cells	Demonstrate the importance of microenvironment on the composition of HCC organoids	(213)
Healthy liver resections derived organoids	The organoid can identify different tumor tissue and subtypes and preserves the histological architecture, gene expression and genomic landscape of the original tumor	Identify the ERK inhibitor SCH772984 as a potential drug for HCC	(214)
Needle biopsies from HCC patients	HCC organoids maintain the genomic features of their originating tumors during long-term culturing for up to 32 weeks.	Illustrate the function of testing sensitivity to sorafenib and providing a tool for developing tailored therapies.	(215)
Reprogrammed human hepatocytes (hiHeps)	We employed hiHeps to establish an improved organoid model possessing liver architecture and function	Prove c-Myc-induced human HCC initiation was associated with the alteration of MAMs^*^ and RAS-induced lineage conversion from hepatocytes to ICC^*^ cells can be prevented by the combined inhibition of Notch and JAK–STAT	(216)
Distinct regions of liver tumor	A total of 27 liver cancer lines were established and 129 cancer drugs were tested	Demonstrate the usage of cancer organoid drug testing as part of a drug discovery pipeline	(217)

### Contributions to therapeutic resistance in HCC microenvironment

**Figure 2:**
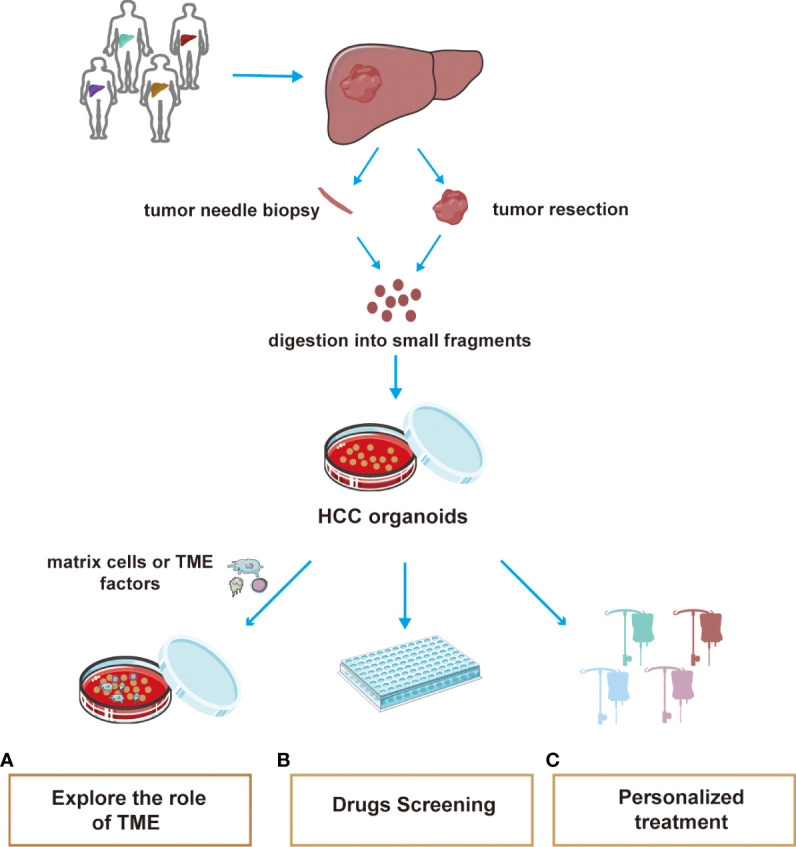
A summary of the main applications of HCC organoid. The tissue used in organoid culture of hepatocellular carcinoma is mostly obtained by pathological biopsy and surgical resection. Tissues were cultured for 3D using special media. At present, the application of organoids mainly includes three aspects: **(A)** Co-culture of HCC organoids with matrix cells or extracellular cytokines helps explore the effects of the TME on HCC progression or therapeutic resistance powerfully. **(B)** Efficient culture organoids are suitable for high-throughput drug screening **(C)** Organoid drug screening helps to develop individualized treatment regimen for different patients.

The most common method for organoid culture of liver cancer is to transport liver cancer patient tissues back to the laboratory for 3D culture (218). Organoid structures contain a variety of specific cell types and their spatial structures are similar to those of their corresponding tumor tissues. With improvements in physiological modeling methods, organoid culture techniques can be combined with *in vitro* TME techniques to maintain diverse cell populations. The cultivation system of organoids simulating the tumor microenvironment, screening for sensitive clinical drugs, and achieving precise individualized medicine are three aspects of great significance in exploring clinical drug resistance in patients with HCC (**Figure 2**).

Co-culture of HCC organoids with matrix cells or extracellular cytokines helps explain the effects of the TME on HCC growth. In 2017, Wang et al. constructed HCC organoids and organoids with fibroblasts and endothelial cells. Immunofluorescence results showed that the addition of non-parenchymal cells greatly enhanced the expression of EMT-related molecules (MMP9, vimentin, and TGF-B), tumor-related inflammatory factors (TNF-α, CXCL12, and CXCR4), and neo-angiogenesis-related markers (VEGF, VEGFR2, and HIF-α), which are important factors in the tumor microenvironment for tumor growth and drug resistance (213). This was a very successful integration of microenvironment and organoid models. Furthermore, to demonstrate the effect of FSTL1 secreted by fibroblasts in the HCC microenvironment on drug resistance in HCC, Loh’s team constructed the patient-derived HCC organoid model and used FSTL1 overexpressing conditioned medium to culture the organoids. The results showed that FSTL1 enhanced the ability of hepatocytes to resist sorafenib through the AKT/mTOR/4EBP1/c-MYC signaling axis. In the mouse model, the administration of FSTL1 antibody enhanced the sensitivity of sorafenib, demonstrating the accuracy of *in vitro* organoid assays (219). Although research on the organoid-TME model in liver cancer has not been popularized, there is no doubt that this model provides novel ideas and important potential for the study of the mechanisms of drug resistance in the HCC microenvironment.

The organoid model has a short culture cycle and high success rate, which is conducive to high-throughput drug screening (220). Broutier et al. found that cultured HCC organoids were insensitive to BRAF and/or MEK inhibitors. However, in a patient-derived organoid, SCH772984 selectively inhibited ERK phosphorylation and significantly inhibited tumorigenesis (214). Numerous studies have demonstrated that the TME is greatly involved in the development of drug resistance in patients with HCC. Therefore, when drug resistance occurs in clinical patients, organoids can be cultured *in vitro* by biopsy puncture or surgical resection and then the drug-sensitive patients can be screened in batches.

Personalized treatment can be achieved through organoid drug screening. Recently, some patient-derived organoids from different individuals cultured with hydrogel capsules, which can simulate the TME of liver cancer, were tested for sensitivity to cabazitaxel, oxaliplatin, and sorafenib. As a result, sensitivity was found to differ among individuals. Magnetic resonance imaging and biochemical examinations were used at later follow-up to test the cost-effectiveness of organoid screening drugs for patients (221). Therefore, it is a promising platform for realizing economical personalized treatment. Currently, several preclinical studies on HCC organoids are underway (NCT05384184, NCT02436564, and NCT02718235).

## Conclusion and future directions

In this review, we have mainly discussed the relationship between the microenvironment and drug resistance associated with liver cancer treatment from various aspects and summarized the targets and directions of future treatment of liver cancer. Currently, the standard treatments for hepatocellular carcinoma remain surgical resection, radiofrequency ablation, TACE, and systemic therapy. Due to drug resistance and high recurrence rates, most patients cannot benefit from existing therapies. Especially in cases of HCC with high heterogeneity, it is even more difficult to precisely target biomarkers. Accumulating evidence has shown that the interaction between tumor cells and the tumor microenvironment is crucial for tumor cell survival, proliferation, acquisition of stem cell characteristics, invasion, metastasis, and drug resistance. Therapies targeting the TME represent a breakthrough in addressing therapeutic resistance. Accordingly, most researchers have suggested that combination therapy may be the mainstay treatment for HCC in the future, including ICI, lenvatinib, sorafenib, and targeted therapy.

However, due to the heterogeneous subpopulations that develop during the process of tumor progression, some significant markers such as TERT, TP53, ARID2, ARID1A, and the WNT signaling regulator CTNNB1 cannot be targeted efficiently, which reduces the utility of the predictor from a treatment perspective. Moreover, some drugs perform efficiently *in vivo* and *in vitro* but do not work in clinical settings. These facts highlight the importance of accurate preclinical model prediction. In recent years, human-derived organoids have made great progress in many cancers, which supplements the lack of microenvironmental influence and anthropogenic models compared to traditional 2D and mouse xenograft models. Currently, we have identified heterogeneous stem cell populations and tested hundreds of drugs developed using HCC organoids. Although more time and resources are required to culture organoids *in vitro* than cancer cell lines, the development potential of organoids is substantial. The use of more accurate 3D models to explore microenvironment-targeted therapy is a promising prospect for hepatocellular carcinoma.

## Author contributions

YQ designed this study and carefully reviewed the manuscript. JZ and HH participated in the design and wrote the first manuscript. JZ and LW were mainly responsible for the design of the images. WW and MY were involved in the process of document collection and review. All authors contributed to the article and approved the submitted version.

## Funding

The study was supported by National Natural Science Foundation of China (no. 81872264).

## Acknowledgments

Thanks to Peiming Yan and Daidi Zhang for their support during this study.

## Conflict of interest

The authors declare that the research was conducted in the absence of any commercial or financial relationships that could be construed as a potential conflict of interest.

## Publisher’s note

All claims expressed in this article are solely those of the authors and do not necessarily represent those of their affiliated organizations, or those of the publisher, the editors and the reviewers. Any product that may be evaluated in this article, or claim that may be made by its manufacturer, is not guaranteed or endorsed by the publisher.

## References

[1] Li L Wang H. Heterogeneity of liver cancer and personalized therapy. Cancer Lett (2016) 379(2):191–7. doi: 10.1016/j.canlet.2015.07.018 26213370

[2] Sung H Ferlay J Siegel RL Laversanne M Soerjomataram I Jemal A. Global cancer statistics 2020: Globocan estimates of incidence and mortality worldwide for 36 cancers in 185 countries. CA: Cancer J Clin (2021) 71(3):209–49. doi: 10.3322/caac.21660 33538338

[3] Sia D Villanueva A Friedman SL Llovet JM. Liver cancer cell of origin, molecular class, and effects on patient prognosis. Gastroenterology (2017) 152(4):745–61. doi: 10.1053/j.gastro.2016.11.048 PMC12160040 28043904

[4] Anwanwan D Singh SK Singh S Saikam V Singh R. Challenges in liver cancer and possible treatment approaches. Biochim Biophys Acta Rev Cancer (2020) 1873(1):188314. doi: 10.1016/j.bbcan.2019.188314 31682895 PMC6981221

[5] Wang CI Chu PM Chen YL Lin YH Chen CY. Chemotherapeutic drug-regulated cytokines might influence therapeutic efficacy in hcc. Int J Mol Sci (2021) 22(24):13627. doi: 10.3390/ijms222413627 34948424 PMC8707970

[6] Llovet JM Castet F Heikenwalder M Maini MK Mazzaferro V Pinato DJ. Immunotherapies for hepatocellular carcinoma. Nat Rev Clin Oncol (2022) 19(3):151–72. doi: 10.1038/s41571-021-00573-2 34764464

[7] Bukowski K Kciuk M Kontek R. Mechanisms of multidrug resistance in cancer chemotherapy. Int J Mol Sci (2020) 21(9):3233. doi: 10.3390/ijms21093233 32370233 PMC7247559

[8] Li M Wang X Wang Y Bao S Chang Q Liu L. Strategies for remodeling the tumor microenvironment using active ingredients of ginseng-a promising approach for cancer therapy. Front Pharmacol (2021) 12:797634. doi: 10.3389/fphar.2021.797634 35002732 PMC8727883

[9] Seebacher NA Krchniakova M Stacy AE Skoda J Jansson PJ. Tumour microenvironment stress promotes the development of drug resistance. Antioxidants (Basel Switzerland) (2021) 10(11):1801. doi: 10.3390/antiox10111801 34829672 PMC8615091

[10] Balaji S Kim U Muthukkaruppan V Vanniarajan A. Emerging role of tumor microenvironment derived exosomes in therapeutic resistance and metastasis through epithelial-to-Mesenchymal transition. Life Sci (2021) 280:119750. doi: 10.1016/j.lfs.2021.119750 34171378

[11] Paget S. The distribution of secondary growths in cancer of the breast. 1889. Cancer metastasis Rev (1989) 8(2):98–101. doi: 10.1016/S0140-6736(00)49915-0 2673568

[12] Barry AE Baldeosingh R Lamm R Patel K Zhang K Dominguez DA. Hepatic stellate cells and hepatocarcinogenesis. Front Cell Dev Biol (2020) 8:709. doi: 10.3389/fcell.2020.00709 32850829 PMC7419619

[13] Novikova MV Khromova NV Kopnin PB. Components of the hepatocellular carcinoma microenvironment and their role in tumor progression. Biochem Biokhimiia (2017) 82(8):861–73. doi: 10.1134/s0006297917080016 28941454

[14] Bao MH Wong CC. Hypoxia, metabolic reprogramming, and drug resistance in liver cancer. Cells (2021) 10(7):1715. doi: 10.3390/cells10071715 34359884 PMC8304710

[15] Craig AJ von Felden J Garcia-Lezana T Sarcognato S Villanueva A. Tumour evolution in hepatocellular carcinoma. Nat Rev Gastroenterol Hepatol (2020) 17(3):139–52. doi: 10.1038/s41575-019-0229-4 31792430

[16] Finn RS Qin S Ikeda M Galle PR Ducreux M Kim TY. Atezolizumab plus bevacizumab in unresectable hepatocellular carcinoma. New Engl J Med (2020) 382(20):1894–905. doi: 10.1056/NEJMoa1915745 32402160

[17] Qin S Li A Yi M Yu S Zhang M Wu K. Recent advances on anti-angiogenesis receptor tyrosine kinase inhibitors in cancer therapy. J Hematol Oncol (2019) 12(1):27. doi: 10.1186/s13045-019-0718-5 30866992 PMC6417086

[18] Ioannidou E Moschetta M Shah S Parker JS Ozturk MA Pappas-Gogos G. Angiogenesis and anti-angiogenic treatment in prostate cancer: Mechanisms of action and molecular targets. Int J Mol Sci (2021) 22(18):9926. doi: 10.3390/ijms22189926 34576107 PMC8472415

[19] Folkman J. Tumor angiogenesis: Therapeutic implications. New Engl J Med (1971) 285(21):1182–6. doi: 10.1056/nejm197111182852108 4938153

[20] Rinaldi L Vetrano E Rinaldi B Galiero R Caturano A Salvatore T. Hcc and molecular targeting therapies: Back to the future. Biomedicines (2021) 9(10):1345. doi: 10.3390/biomedicines9101345 34680462 PMC8533575

[21] Carmeliet P Moons L Luttun A Vincenti V Compernolle V De Mol M. Synergism between vascular endothelial growth factor and placental growth factor contributes to angiogenesis and plasma extravasation in pathological conditions. Nat Med (2001) 7(5):575–83. doi: 10.1038/87904 11329059

[22] Fischer C Jonckx B Mazzone M Zacchigna S Loges S Pattarini L. Anti-plgf inhibits growth of Vegf(R)-Inhibitor-Resistant tumors without affecting healthy vessels. Cell (2007) 131(3):463–75. doi: 10.1016/j.cell.2007.08.038 17981115

[23] Willett CG Duda DG di Tomaso E Boucher Y Ancukiewicz M Sahani DV. Efficacy, safety, and biomarkers of neoadjuvant bevacizumab, radiation therapy, and fluorouracil in rectal cancer: A multidisciplinary phase ii study. J Clin oncology: Off J Am Soc Clin Oncol (2009) 27(18):3020–6. doi: 10.1200/jco.2008.21.1771 PMC2702234 19470921

[24] Xu HX Zhu XD Zhuang PY Zhang JB Zhang W Kong LQ. Expression and prognostic significance of placental growth factor in hepatocellular carcinoma and peritumoral liver tissue. Int J Cancer (2011) 128(7):1559–69. doi: 10.1002/ijc.25492 20521248

[25] Maniotis A Folberg R Hess A Seftor E Gardner L Pe’er J. Vascular channel formation by human melanoma cells in vivo and in vitro: Vasculogenic mimicry. The American Journal of Pathology (1999) 155(3):739–52. doi: 10.1016/s0002-9440(10)65173-5 PMC1866899 10487832

[26] Belotti D Pinessi D Taraboletti G. Alternative vascularization mechanisms in tumor resistance to therapy. Cancers (2021) 13(8):1912. doi: 10.3390/cancers13081912 33921099 PMC8071410

[27] Hendrix MJ Seftor EA Seftor RE Chao JT Chien DS Chu YW. Tumor cell vascular mimicry: Novel targeting opportunity in melanoma. Pharmacol Ther (2016) 159:83–92. doi: 10.1016/j.pharmthera.2016.01.006 26808163 PMC4779708

[28] Luo Q Wang J Zhao W Peng Z Liu X Li B. Vasculogenic mimicry in carcinogenesis and clinical applications. J Hematol Oncol (2020) 13(1):19. doi: 10.1186/s13045-020-00858-6 32169087 PMC7071697

[29] Mao Y Zhu L Huang Z Luo C Zhou T Li L. Stem-like tumor cells involved in heterogeneous vasculogenesis in breast cancer. Endocrine-related Cancer (2020) 27(1):23–39. doi: 10.1530/erc-19-0054 31705798

[30] Treps L Faure S Clere NJP. Vasculogenic mimicry, a complex and devious process favoring tumorigenesis - interest in making it a therapeutic target. Pharmacology & Therapeutics (2021) 223:107805. doi: 10.1016/j.pharmthera.2021.107805. therapeutics. 33465401

[31] Xu J Zhang Y Wang Y Tao X Cheng L Wu S. Correlation of Kai1, Cd133 and vasculogenic mimicry with the prediction of metastasis and prognosis in hepatocellular carcinoma. Int J Clin Exp Pathol (2018) 11(7):3638–46. PMC6962867 31949744

[32] Shi Y Shang J Li Y Zhong D Zhang Z Yang Q. Itga5 and Itgb1 contribute to sorafenib resistance by promoting vasculogenic mimicry formation in hepatocellular carcinoma. Cancer Med (2022). doi: 10.1002/cam4.5110 PMC9939139 35946175

[33] Wang M Zhao X Zhu D Liu T Liang X Liu F. Hif-1α promoted vasculogenic mimicry formation in hepatocellular carcinoma through Loxl2 up-regulation in hypoxic tumor microenvironment. J Exp Clin Cancer research: CR (2017) 36(1):60. doi: 10.1186/s13046-017-0533-1 28449718 PMC5408450

[34] Qiao K Liu Y Xu Z Zhang H Zhang H Zhang C. Rna M6a methylation promotes the formation of vasculogenic mimicry in hepatocellular carcinoma *Via* hippo pathway. Angiogenesis (2021) 24(1):83–96. doi: 10.1007/s10456-020-09744-8 32920668

[35] Huang XY Huang ZL Huang J Xu B Huang XY Xu YH. Exosomal circrna-100338 promotes hepatocellular carcinoma metastasis *Via* enhancing invasiveness and angiogenesis. J Exp Clin Cancer research: CR (2020) 39(1):20. doi: 10.1186/s13046-020-1529-9 31973767 PMC6979009

[36] Cheng R Wang B Cai XR Chen ZS Du Q Zhou LY. Cd276 promotes vasculogenic mimicry formation in hepatocellular carcinoma *Via* the Pi3k/Akt/Mmps pathway. OncoTargets Ther (2020) 13:11485–98. doi: 10.2147/ott.S271891 PMC7667184 33204103

[37] Li X Sun B Zhao X An J Zhang Y Gu Q. Function of Bmp4 in the formation of vasculogenic mimicry in hepatocellular carcinoma. J Cancer (2020) 11(9):2560–71. doi: 10.7150/jca.40558 PMC7066000 32201526

[38] Qu B Sheng G Guo L Yu F Chen G Lu Q. Mig7 is involved in vasculogenic mimicry formation rendering invasion and metastasis in hepatocellular carcinoma. Oncol Rep (2018) 39(2):679–86. doi: 10.3892/or.2017.6138 29251318

[39] Zhao X Sun B Liu T Shao B Sun R Zhu D. Long noncoding rna N339260 promotes vasculogenic mimicry and cancer stem cell development in hepatocellular carcinoma. Cancer Sci (2018) 109(10):3197–208. doi: 10.1111/cas.13740 PMC6172069 30022558

[40] Zhang JG Zhang DD Liu Y Hu JN Zhang X Li L. Rhoc/Rock2 promotes vasculogenic mimicry formation primarily through Erk/Mmps in hepatocellular carcinoma. Biochim Biophys Acta Mol basis Dis (2019) 1865(6):1113–25. doi: 10.1016/j.bbadis.2018.12.007 30779947

[41] Sun D Sun B Liu T Zhao X Che N Gu Q. Slug promoted vasculogenic mimicry in hepatocellular carcinoma. J Cell Mol Med (2013) 17(8):1038–47. doi: 10.1111/jcmm.12087 PMC3780534 23815612

[42] Liu W Xu G Ma J Jia W Li J Chen K. Osteopontin as a key mediator for vasculogenic mimicry in hepatocellular carcinoma. Tohoku J Exp Med (2011) 224(1):29–39. doi: 10.1620/tjem.224.29 21512310

[43] Meng J Chen S Lei YY Han JX Zhong WL Wang XR. Hsp90B promotes aggressive vasculogenic mimicry *Via* epithelial-mesenchymal transition in hepatocellular carcinoma. Oncogene (2019) 38(2):228–43. doi: 10.1038/s41388-018-0428-4 30087438

[44] Jue C Lin C Zhisheng Z Yayun Q Feng J Min Z. Notch1 promotes vasculogenic mimicry in hepatocellular carcinoma by inducing emt signaling. Oncotarget (2017) 8(2):2501–13. doi: 10.18632/oncotarget.12388 PMC5356819 27705934

[45] Yang Z Sun B Li Y Zhao X Zhao X Gu Q. Zeb2 promotes vasculogenic mimicry by tgf-B1 induced epithelial-to-Mesenchymal transition in hepatocellular carcinoma. Exp Mol Pathol (2015) 98(3):352–9. doi: 10.1016/j.yexmp.2015.03.030 25818166

[46] Sun T Zhao N Zhao XL Gu Q Zhang SW Che N. Expression and functional significance of Twist1 in hepatocellular carcinoma: Its role in vasculogenic mimicry. Hepatol (Baltimore Md) (2010) 51(2):545–56. doi: 10.1002/hep.23311 19957372

[47] Donnem T Hu J Ferguson M Adighibe O Snell C Harris AL. Vessel Co-option in primary human tumors and metastases: An obstacle to effective anti-angiogenic treatment? Cancer Med (2013) 2(4):427–36. doi: 10.1002/cam4.105 PMC3799277 24156015

[48] Kuczynski EA Yin M Bar-Zion A Lee CR Butz H Man S. Co-Option of liver vessels and not sprouting angiogenesis drives acquired sorafenib resistance in hepatocellular carcinoma. J Natl Cancer Institute (2016) 108(8):djw030. doi: 10.1093/jnci/djw030 PMC5017954 27059374

[49] Kuczynski EA Kerbel RS. Implications of vessel Co-option in sorafenib-resistant hepatocellular carcinoma. Chin J Cancer (2016) 35(1):97. doi: 10.1186/s40880-016-0162-7 27887628 PMC5124233

[50] Rada M Tsamchoe M Kapelanski-Lamoureux A Hassan N Bloom J Petrillo S. Cancer cells promote phenotypic alterations in hepatocytes at the edge of cancer cell nests to facilitate vessel Co-option establishment in colorectal cancer liver metastases. Cancers (2022) 14(5):1318. doi: 10.3390/cancers14051318 35267627 PMC8909291

[51] Wang M Ren S Bi Z Zhang L Cui M Sun R. Myricetin reverses epithelial-endothelial transition and inhibits vasculogenic mimicry and angiogenesis of hepatocellular carcinoma by directly targeting Par1. Phytotherapy research: PTR (2022) 36 :1807–21. doi: 10.1002/ptr.7427 35229382

[52] Zhang X Zhang JG Mu W Zhou HM Liu GL Li Q. The role of daurisoline treatment in hepatocellular carcinoma: Inhibiting vasculogenic mimicry formation and enhancing sensitivity to sorafenib. Phytomedicine: Int J phytotherapy phytopharmacology (2021) 92:153740. doi: 10.1016/j.phymed.2021.153740 34600176

[53] Lv C Wang S Lin L Wang C Zeng K Meng Y. Usp14 maintains Hif1-A; stabilization *Via* its deubiquitination activity in hepatocellular carcinoma. Cell Death Dis (2021) 12(9):803. doi: 10.1038/s41419-021-04089-6 34420039 PMC8380251

[54] Zhang N Zhang S Wu W Lu W Jiang M Zheng N. Regorafenib inhibits migration, invasion, and vasculogenic mimicry of hepatocellular carcinoma *Via* targeting Id1-mediated emt. Mol carcinogenesis (2021) 60(2):151–63. doi: 10.1002/mc.23279 33428809

[55] Bao S Jin S Wang C Tu P Hu K Lu J. Androgen receptor suppresses vasculogenic mimicry in hepatocellular carcinoma *Via* Circrna7/Mirna7-5p/Ve-Cadherin/Notch4 signalling. J Cell Mol Med (2020) 24(23):14110–20. doi: 10.1111/jcmm.16022 PMC7754040 33118329

[56] Zhang X Zhang J Zhou H Liu G Li Q. Rho kinase mediates transforming growth factor-B1-Induced vasculogenic mimicry formation: Involvement of the epithelial-mesenchymal transition and cancer stemness activity. Acta Biochim Biophys Sin (2020) 52(4):411–20. doi: 10.1093/abbs/gmaa014 32296834

[57] Chen Q Lin W Yin Z Zou Y Liang S Ruan S. Melittin inhibits hypoxia-induced vasculogenic mimicry formation and epithelial-mesenchymal transition through suppression of hif-1α/Akt pathway in liver cancer. Evidence-Based complementary Altern medicine: eCAM (2019) 2019:9602935. doi: 10.1155/2019/9602935 PMC6463627 31057657

[58] An H Lin J Sun H Xu L Su J He C. [Biejiajian pills inhibits hepatoma carcinoma cell vasculogenic mimicry by suppressing Rhoa/Rock signaling pathway]. Nan fang yi ke da xue xue bao = J South Med Univ (2018) 38(8):997–1001. doi: 10.3969/j.issn.1673-4254.2018.08.16 PMC6744031 30187871

[59] Xiao T Zhong W Zhao J Qian B Liu H Chen S. Polyphyllin I suppresses the formation of vasculogenic mimicry *Via* Twist1/Ve-cadherin pathway. Cell Death Dis (2018) 9(9):906. doi: 10.1038/s41419-018-0902-5 30185783 PMC6125388

[60] Zhang F Zhang CM Li S Wang KK Guo BB Fu Y. Low dosage of arsenic trioxide inhibits vasculogenic mimicry in hepatoblastoma without cell apoptosis. Mol Med Rep (2018) 17(1):1573–82. doi: 10.3892/mmr.2017.8046 PMC5780096 29138840

[61] Hao X Sun G Zhang Y Kong X Rong D Song J. Targeting immune cells in the tumor microenvironment of hcc: New opportunities and challenges. Front Cell Dev Biol (2021) 9:775462. doi: 10.3389/fcell.2021.775462 34869376 PMC8633569

[62] Gao Q Qiu SJ Fan J Zhou J Wang XY Xiao YS. Intratumoral balance of regulatory and cytotoxic T cells is associated with prognosis of hepatocellular carcinoma after resection. J Clin oncology: Off J Am Soc Clin Oncol (2007) 25(18):2586–93. doi: 10.1200/jco.2006.09.4565 17577038

[63] Li Z Li N Li F Zhou Z Sang J Chen Y. Immune checkpoint proteins pd-1 and Tim-3 are both highly expressed in liver tissues and correlate with their gene polymorphisms in patients with hbv-related hepatocellular carcinoma. Medicine (2016) 95(52):e5749. doi: 10.1097/md.0000000000005749 28033288 PMC5207584

[64] Chiu DK Yuen VW Cheu JW Wei LL Ting V Fehlings M. Hepatocellular carcinoma cells up-regulate Pvrl1, stabilizing pvr and inhibiting the cytotoxic T-cell response *Via* tigit to mediate tumor resistance to Pd1 inhibitors in mice. Gastroenterology (2020) 159(2):609–23. doi: 10.1053/j.gastro.2020.03.074 32275969

[65] Karabicici M Azbazdar Y Ozhan G Senturk S Firtina Karagonlar Z Erdal E. Changes in wnt and tgf-B signaling mediate the development of regorafenib resistance in hepatocellular carcinoma cell line Huh7. Front Cell Dev Biol (2021) 9:639779. doi: 10.3389/fcell.2021.639779 34458250 PMC8386122

[66] Shrestha R Prithviraj P Bridle KR Crawford DHG Jayachandran A. Combined inhibition of tgf-B1-Induced emt and pd-L1 silencing re-sensitizes hepatocellular carcinoma to sorafenib treatment. J Clin Med (2021) 10(9):1889. doi: 10.3390/jcm10091889 33925488 PMC8123871

[67] Arce-Sillas A Álvarez-Luquín DD Tamaya-Domínguez B Gomez-Fuentes S Trejo-García A Melo-Salas M. Regulatory T cells: Molecular actions on effector cells in immune regulation. J Immunol Res (2016) 2016:1720827. doi: 10.1155/2016/1720827 27298831 PMC4889823

[68] Augustin RC Leone RD Naing A Fong L Bao R Luke JJ. Next steps for clinical translation of adenosine pathway inhibition in cancer immunotherapy. J immunotherapy Cancer (2022) 10(2):e004089. doi: 10.1136/jitc-2021-004089 PMC8830302 35135866

[69] Bengsch B Ohtani T Herati RS Bovenschen N Chang KM Wherry EJ. Deep immune profiling by mass cytometry links human T and nk cell differentiation and cytotoxic molecule expression patterns. J Immunol Methods (2018) 453:3–10. doi: 10.1016/j.jim.2017.03.009 28322863 PMC5605401

[70] Gao Y You M Fu J Tian M Zhong X Du C. Intratumoral stem-like Ccr4+ regulatory T cells orchestrate the immunosuppressive microenvironment in hcc associated with hepatitis b. J Hepatol (2022) 76(1):148–59. doi: 10.1016/j.jhep.2021.08.029 34689996

[71] Rodriguez PC Quiceno DG Ochoa AC. L-arginine availability regulates T-lymphocyte cell-cycle progression. Blood (2007) 109(4):1568–73. doi: 10.1182/blood-2006-06-031856 PMC1794048 17023580

[72] Levring TB Kongsbak M Rode AK Woetmann A Ødum N Bonefeld CM. Human Cd4+ T cells require exogenous cystine for glutathione and DNA synthesis. Oncotarget (2015) 6(26):21853–64. doi: 10.18632/oncotarget.5213 PMC4673131 26392411

[73] Ma T Renz BW Ilmer M Koch D Yang Y Werner J. Myeloid-derived suppressor cells in solid tumors. Cells (2022) 11(2):310. doi: 10.3390/cells11020310 35053426 PMC8774531

[74] Lu T Gabrilovich D. Molecular pathways: Tumor-infiltrating myeloid cells and reactive oxygen species in regulation of tumor microenvironment. Clinical Cancer Research (2012) 18(18):4877–82. doi: 10.1158/1078-0432.Ccr-11-2939. JCcraojotAAfCR. PMC3445728 22718858

[75] Kapanadze T Gamrekelashvili J Ma C Chan C Zhao F Hewitt S. Regulation of accumulation and function of myeloid derived suppressor cells in different murine models of hepatocellular carcinoma. J Hepatol (2013) 59(5):1007–13. doi: 10.1016/j.jhep.2013.06.010 PMC3805787 23796475

[76] Deng X Li X Guo X Lu Y Xie Y Huang X. Myeloid-derived suppressor cells promote tumor growth and sorafenib resistance by inducing Fgf1 upregulation and fibrosis. Neoplasia (New York NY) (2022) 28:100788. doi: 10.1016/j.neo.2022.100788 PMC8980488 35378464

[77] Chiu DK Tse AP Xu IM Di Cui J Lai RK Li LL. Hypoxia inducible factor hif-1 promotes myeloid-derived suppressor cells accumulation through Entpd2/Cd39l1 in hepatocellular carcinoma. Nat Commun (2017) 8(1):517. doi: 10.1038/s41467-017-00530-7 28894087 PMC5593860

[78] Eckert I Ribechini E Lutz MB. *In vitro* generation of murine myeloid-derived suppressor cells, analysis of markers, developmental commitment, and function. Methods Mol Biol (Clifton NJ) (2021) 2236:99–114. doi: 10.1007/978-1-0716-1060-2_10 33237544

[79] Shojaei F Wu X Malik AK Zhong C Baldwin ME Schanz S. Tumor refractoriness to anti-vegf treatment is mediated by Cd11b+Gr1+ myeloid cells. Nat Biotechnol (2007) 25(8):911–20. doi: 10.1038/nbt1323 17664940

[80] Xu M Zhao Z Song J Lan X Lu S Chen M. Interactions between interleukin-6 and myeloid-derived suppressor cells drive the chemoresistant phenotype of hepatocellular cancer. Exp Cell Res (2017) 351(2):142–9. doi: 10.1016/j.yexcr.2017.01.008 28109867

[81] Erin N Grahovac J Brozovic A Efferth T. Tumor microenvironment and epithelial mesenchymal transition as targets to overcome tumor multidrug resistance. Drug resistance updates: Rev commentaries antimicrobial Anticancer chemotherapy (2020) 53:100715. doi: 10.1016/j.drup.2020.100715 32679188

[82] Kang D Han Z Oh GH Joo Y Choi HJ Song JJ. Down-regulation of tgf-B expression sensitizes the resistance of hepatocellular carcinoma cells to sorafenib. Yonsei Med J (2017) 58(5):899–909. doi: 10.3349/ymj.2017.58.5.899 28792132 PMC5552643

[83] Zhou J Liu M Sun H Feng Y Xu L Chan AWH. Hepatoma-intrinsic ccrk inhibition diminishes myeloid-derived suppressor cell immunosuppression and enhances immune-checkpoint blockade efficacy. Gut (2018) 67(5):931–44. doi: 10.1136/gutjnl-2017-314032 PMC5961939 28939663

[84] Li YM Liu ZY Wang JC Yu JM Li ZC Yang HJ. Receptor-interacting protein kinase 3 deficiency recruits myeloid-derived suppressor cells to hepatocellular carcinoma through the chemokine (C-X-C motif) ligand 1-chemokine (C-X-C motif) receptor 2 axis. Hepatol (Baltimore Md) (2019) 70(5):1564–81. doi: 10.1002/hep.30676 PMC6900048 31021443

[85] Yeung OW Lo CM Ling CC Qi X Geng W Li CX. Alternatively activated (M2) macrophages promote tumour growth and invasiveness in hepatocellular carcinoma. J Hepatol (2015) 62(3):607–16. doi: 10.1016/j.jhep.2014.10.029 25450711

[86] Sumitomo R Hirai T Fujita M Murakami H Otake Y Huang CL. M2 tumor-associated macrophages promote tumor progression in non-Small-Cell lung cancer. Exp Ther Med (2019) 18(6):4490–8. doi: 10.3892/etm.2019.8068 PMC6862535 31777551

[87] Kakoschky B Pleli T Schmithals C Zeuzem S Brüne B Vogl TJ. Selective targeting of tumor associated macrophages in different tumor models. PloS One (2018) 13(2):e0193015. doi: 10.1371/journal.pone.0193015 29447241 PMC5814016

[88] Dong N Shi X Wang S Gao Y Kuang Z Xie Q. M2 macrophages mediate sorafenib resistance by secreting hgf in a feed-forward manner in hepatocellular carcinoma. Br J Cancer (2019) 121(1):22–33. doi: 10.1038/s41416-019-0482-x 31130723 PMC6738111

[89] Fu XT Song K Zhou J Shi YH Liu WR Shi GM. Tumor-associated macrophages modulate resistance to oxaliplatin *Via* inducing autophagy in hepatocellular carcinoma. Cancer Cell Int (2019) 19:71. doi: 10.1186/s12935-019-0771-8 30962765 PMC6434873

[90] Avila MA Berasain C. Targeting Ccl2/Ccr2 in tumor-infiltrating macrophages: A tool emerging out of the box against hepatocellular carcinoma. Cell Mol Gastroenterol Hepatol (2019) 7(2):293–4. doi: 10.1016/j.jcmgh.2018.11.002 PMC6354282 30529279

[91] Yao W Ba Q Li X Li H Zhang S Yuan Y. A natural Ccr2 antagonist relieves tumor-associated macrophage-mediated immunosuppression to produce a therapeutic effect for liver cancer. EBioMedicine (2017) 22:58–67. doi: 10.1016/j.ebiom.2017.07.014 28754304 PMC5552238

[92] Chen Y Ramjiawan RR Reiberger T Ng MR Hato T Huang Y. Cxcr4 inhibition in tumor microenvironment facilitates anti-programmed death receptor-1 immunotherapy in sorafenib-treated hepatocellular carcinoma in mice. Hepatol (Baltimore Md) (2015) 61(5):1591–602. doi: 10.1002/hep.27665 PMC4406806 25529917

[93] Anderson NR Minutolo NG Gill S Klichinsky M. Macrophage-based approaches for cancer immunotherapy. Cancer Res (2021) 81(5):1201–8. doi: 10.1158/0008-5472.Can-20-2990 33203697

[94] Ao JY Zhu XD Chai ZT Cai H Zhang YY Zhang KZ. Colony-stimulating factor 1 receptor blockade inhibits tumor growth by altering the polarization of tumor-associated macrophages in hepatocellular carcinoma. Mol Cancer Ther (2017) 16(8):1544–54. doi: 10.1158/1535-7163.Mct-16-0866 28572167

[95] Tan HY Wang N Man K Tsao SW Che CM Feng Y. Autophagy-induced Relb/P52 activation mediates tumour-associated macrophage repolarisation and suppression of hepatocellular carcinoma by natural compound baicalin. Cell Death Dis (2015) 6(10):e1942. doi: 10.1038/cddis.2015.271 26492375 PMC4632300

[96] Xu G Feng D Yao Y Li P Sun H Yang H. Listeria-based hepatocellular carcinoma vaccine facilitates anti-Pd-1 therapy by regulating macrophage polarization. Oncogene (2020) 39(7):1429–44. doi: 10.1038/s41388-019-1072-3 31659256

[97] Lu S Gao Y Huang X Wang X. Cantharidin exerts anti-hepatocellular carcinoma by mir-214 modulating macrophage polarization. Int J Biol Sci (2014) 10(4):415–25. doi: 10.7150/ijbs.8002 PMC3979994 24719559

[98] Xu W Cheng Y Guo Y Yao W Qian H. Targeting tumor associated macrophages in hepatocellular carcinoma. Biochem Pharmacol (2022) 199:114990. doi: 10.1016/j.bcp.2022.114990 35288152

[99] Yin Z Ma T Lin Y Lu X Zhang C Chen S. Il-6/Stat3 pathway intermediates M1/M2 macrophage polarization during the development of hepatocellular carcinoma. J Cell Biochem (2018) 119(11):9419–32. doi: 10.1002/jcb.27259 30015355

[100] Sivori S Della Chiesa M Carlomagno S Quatrini L Munari E Vacca P. Inhibitory receptors and checkpoints in human nk cells, implications for the immunotherapy of cancer. Front Immunol (2020) 11:2156. doi: 10.3389/fimmu.2020.02156 33013909 PMC7494755

[101] Konjević GM Vuletić AM Mirjačić Martinović KM Larsen AK Jurišić VB. The role of cytokines in the regulation of nk cells in the tumor environment. Cytokine (2019) 117:30–40. doi: 10.1016/j.cyto.2019.02.001 30784898

[102] Wu Y Kuang DM Pan WD Wan YL Lao XM Wang D. Monocyte/Macrophage-elicited natural killer cell dysfunction in hepatocellular carcinoma is mediated by Cd48/2b4 interactions. Hepatol (Baltimore Md) (2013) 57(3):1107–16. doi: 10.1002/hep.26192 23225218

[103] Piñeiro Fernández J Luddy KA Harmon C O’Farrelly C. Hepatic tumor microenvironments and effects on nk cell phenotype and function. Int J Mol Sci (2019) 20(17):4131. doi: 10.3390/ijms20174131 31450598 PMC6747260

[104] Park DJ Sung PS Kim JH Lee GW Jang JW Jung ES. Epcam-high liver cancer stem cells resist natural killer cell-mediated cytotoxicity by upregulating Ceacam1. J immunotherapy Cancer (2020) 8(1):e000301. doi: 10.1136/jitc-2019-000301 PMC7206970 32221015

[105] Cheung PF Yip CW Ng LW Wong CK Cheung TT Lo CM. Restoration of natural killer activity in hepatocellular carcinoma by treatment with antibody against granulin-epithelin precursor. Oncoimmunology (2015) 4(7):e1016706. doi: 10.1080/2162402x.2015.1016706 26140244 PMC4485783

[106] Fang L Gong J Wang Y Liu R Li Z Wang Z. Mica/B expression is inhibited by unfolded protein response and associated with poor prognosis in human hepatocellular carcinoma. J Exp Clin Cancer research: CR (2014) 33(1):76. doi: 10.1186/s13046-014-0076-7 25228093 PMC4174668

[107] Ren C Ren X Cao D Zhao H Zhai Z Li H. Cnot7 depletion reverses natural killer cell resistance by modulating the tumor immune microenvironment of hepatocellular carcinoma. FEBS Open Bio (2020) 10(5):847–60. doi: 10.1002/2211-5463.12836 PMC7193174 32160402

[108] Bugide S Green MR Wajapeyee N. Inhibition of enhancer of zeste homolog 2 (Ezh2) induces natural killer cell-mediated eradication of hepatocellular carcinoma cells. Proc Natl Acad Sci United States America (2018) 115(15):E3509–e18. doi: 10.1073/pnas.1802691115 PMC5899497 29581297

[109] Xie H Zhang Q Zhou H Zhou J Zhang J Jiang Y. Microrna-889 is downregulated by histone deacetylase inhibitors and confers resistance to natural killer cytotoxicity in hepatocellular carcinoma cells. Cytotechnology (2018) 70(2):513–21. doi: 10.1007/s10616-017-0108-1 PMC5851948 28550492

[110] Oura K Morishita A Tani J Masaki T. Tumor immune microenvironment and immunosuppressive therapy in hepatocellular carcinoma: A review. Int J Mol Sci (2021) 22(11):5801. doi: 10.3390/ijms22115801 34071550 PMC8198390

[111] Graziani G Tentori L Navarra P. Ipilimumab: A novel immunostimulatory monoclonal antibody for the treatment of cancer. Pharmacol Res (2012) 65(1):9–22. doi: 10.1016/j.phrs.2011.09.002 21930211

[112] Guazzelli A Hussain M Krstic-Demonacos M Mutti L. Tremelimumab for the treatment of malignant mesothelioma. Expert Opin Biol Ther (2015) 15(12):1819–29. doi: 10.1517/14712598.2015.1116515 26560442

[113] Wong JSL Kwok GGW Tang V Li BCW Leung R Chiu J. Ipilimumab and Nivolumab/Pembrolizumab in advanced hepatocellular carcinoma refractory to prior immune checkpoint inhibitors. J immunotherapy Cancer (2021) 9(2):e001945. doi: 10.1136/jitc-2020-001945 PMC7875295 33563773

[114] Kelley RK Sangro B Harris W Ikeda M Okusaka T Kang YK. Safety, efficacy, and pharmacodynamics of tremelimumab plus durvalumab for patients with unresectable hepatocellular carcinoma: Randomized expansion of a phase I/Ii study. J Clin oncology: Off J Am Soc Clin Oncol (2021) 39(27):2991–3001. doi: 10.1200/jco.20.03555 PMC8445563 34292792

[115] Son Y Shin NR Kim SH Park SC Lee HJ. Fibrinogen-like protein 1 modulates sorafenib resistance in human hepatocellular carcinoma cells. Int J Mol Sci (2021) 22(10):5330. doi: 10.3390/ijms22105330 34069373 PMC8158706

[116] Wang J Sanmamed MF Datar I Su TT Ji L Sun J. Fibrinogen-like protein 1 is a major immune inhibitory ligand of lag-3. Cell (2019) 176(1-2):334–47.e12. doi: 10.1016/j.cell.2018.11.010 30580966 PMC6365968

[117] Atkinson V Khattak A Haydon A Eastgate M Roy A Prithviraj P. Eftilagimod alpha, a soluble lymphocyte activation gene-3 (Lag-3) protein plus pembrolizumab in patients with metastatic melanoma. J immunotherapy Cancer (2020) 8(2):e001681. doi: 10.1136/jitc-2020-001681 PMC7682474 33219094

[118] He Y Wang Y Zhao S Zhao C Zhou C Hirsch FR. Slag-3 in non-Small-Cell lung cancer patients’ serum. OncoTargets Ther (2018) 11:4781–4. doi: 10.2147/ott.S164178 PMC6097502 30147330

[119] Johnson DB Nixon MJ Wang Y Wang DY Castellanos E Estrada MV. Tumor-specific mhc-ii expression drives a unique pattern of resistance to immunotherapy *Via* lag-3/Fcrl6 engagement. JCI Insight (2018) 3(24):e120360. doi: 10.1172/jci.insight.120360 30568030 PMC6338319

[120] Hendrickson PG Olson M Luetkens T Weston S Han T Atanackovic D. The promise of adoptive cellular immunotherapies in hepatocellular carcinoma. Oncoimmunology (2020) 9(1):1673129. doi: 10.1080/2162402x.2019.1673129 32002284 PMC6959455

[121] Yin J Li N Han Y Xue J Deng Y Shi J. Effect of antiviral treatment with Nucleotide/Nucleoside analogs on postoperative prognosis of hepatitis b virus-related hepatocellular carcinoma: A two-stage longitudinal clinical study. J Clin oncology: Off J Am Soc Clin Oncol (2013) 31(29):3647–55. doi: 10.1200/jco.2012.48.5896 24002499

[122] Gane E Verdon DJ Brooks AE Gaggar A Nguyen AH Subramanian GM. Anti-Pd-1 blockade with nivolumab with and without therapeutic vaccination for virally suppressed chronic hepatitis b: A pilot study. J Hepatol (2019) 71(5):900–7. doi: 10.1016/j.jhep.2019.06.028 31306680

[123] Mazzocca A Dituri F Lupo L Quaranta M Antonaci S Giannelli G. Tumor-secreted lysophostatidic acid accelerates hepatocellular carcinoma progression by promoting differentiation of peritumoral fibroblasts in myofibroblasts. Hepatol (Baltimore Md) (2011) 54(3):920–30. doi: 10.1002/hep.24485 21674557

[124] Chen Y Zheng X Wu C. The role of the tumor microenvironment and treatment strategies in colorectal cancer. Front Immunol (2021) 12:792691. doi: 10.3389/fimmu.2021.792691 34925375 PMC8674693

[125] Zhang J Gu C Song Q Zhu M Xu Y Xiao M. Identifying cancer-associated fibroblasts as emerging targets for hepatocellular carcinoma. Cell bioscience (2020) 10(1):127. doi: 10.1186/s13578-020-00488-y 33292459 PMC7603733

[126] Chandra Jena B Sarkar S Rout L Mandal M. The transformation of cancer-associated fibroblasts: Current perspectives on the role of tgf-B in caf mediated tumor progression and therapeutic resistance. Cancer Lett (2021) 520:222–32. doi: 10.1016/j.canlet.2021.08.002 34363903

[127] Wang Z Liu J Huang H Ye M Li X Wu R. Metastasis-associated fibroblasts: An emerging target for metastatic cancer. Biomarker Research (2021) 9(1):47. doi: 10.1186/s40364-021-00305-9 34112258 PMC8194104

[128] Mhaidly R Mechta-Grigoriou F. Role of cancer-associated fibroblast subpopulations in immune infiltration, as a new means of treatment in cancer. Immunological Reviews (2021) 302(1):259–72. doi: 10.1111/imr.12978 PMC8360036 34013544

[129] Peng H Xue R Ju Z Qiu J Wang J Yan W. Cancer-associated fibroblasts enhance the chemoresistance of Cd73(+) hepatocellular carcinoma cancer cells *Via* hgf-Met-Erk1/2 pathway. Ann Trans Med (2020) 8(14):856. doi: 10.21037/atm-20-1038 PMC7396767 32793700

[130] Ni Y Soliman A Joehlin-Price A Rose PG Vlad A Edwards RP. High tgf-B signature predicts immunotherapy resistance in gynecologic cancer patients treated with immune checkpoint inhibition. NPJ Precis Oncol (2021) 5(1):101. doi: 10.1038/s41698-021-00242-8 34921236 PMC8683510

[131] Cheng B Ding K Chen P Ji J Luo T Guo X. Anti-Pd-L1/Tgf-Br fusion protein (Shr-1701) overcomes disrupted lymphocyte recovery-induced resistance to pd-1/Pd-L1 inhibitors in lung cancer. Cancer Commun (London England) (2022) 42(1):17–36. doi: 10.1002/cac2.12244 PMC8753312 34981670

[132] Huang K Zhang X Hao Y Feng R Wang H Shu Z. Hypoxia tumor microenvironment activates Gli2 through hif-1α and tgf-B2 to promote chemotherapy resistance of colorectal cancer. Comput Math Methods Med (2022) 2022:2032895. doi: 10.1155/2022/2032895 35186110 PMC8853797

[133] Liu J Yang X Liang Q Yu Y Shen X Sun G. Valproic acid overcomes sorafenib resistance by reducing the migration of Jagged2-mediated Notch1 signaling pathway in hepatocellular carcinoma cells. Int J Biochem Cell Biol (2020) 126:105820. doi: 10.1016/j.biocel.2020.105820 32750425

[134] Liu H Shen J Lu K. Il-6 and pd-L1 blockade combination inhibits hepatocellular carcinoma cancer development in mouse model. Biochem Biophys Res Commun (2017) 486(2):239–44. doi: 10.1016/j.bbrc.2017.02.128 28254435

[135] Gao L Morine Y Yamada S Saito Y Ikemoto T Tokuda K. The Baff/NfKb axis is crucial to interactions between sorafenib-resistant hcc cells and cancer-associated fibroblasts. Cancer Sci (2021) 112(9):3545–54. doi: 10.1111/cas.15041 PMC8409310 34159680

[136] Lau EY Lo J Cheng BY Ma MK Lee JM Ng JK. Cancer-associated fibroblasts regulate tumor-initiating cell plasticity in hepatocellular carcinoma through c-Met/Fra1/Hey1 signaling. Cell Rep (2016) 15(6):1175–89. doi: 10.1016/j.celrep.2016.04.019 27134167

[137] Xiong S Wang R Chen Q Luo J Wang J Zhao Z. Cancer-associated fibroblasts promote stem cell-like properties of hepatocellular carcinoma cells through il-6/Stat3/Notch signaling. Am J Cancer Res (2018) 8(2):302–16. PMC5835697 29511600

[138] Li Y Wang R Xiong S Wang X Zhao Z Bai S. Cancer-associated fibroblasts promote the stemness of Cd24(+) liver cells *Via* paracrine signaling. J Mol Med (Berlin Germany) (2019) 97(2):243–55. doi: 10.1007/s00109-018-1731-9 30564864

[139] Sun L Wang Y Wang L Yao B Chen T Li Q. Resolvin D1 prevents epithelial-mesenchymal transition and reduces the stemness features of hepatocellular carcinoma by inhibiting paracrine of cancer-associated fibroblast-derived comp. J Exp Clin Cancer research: CR (2019) 38(1):170. doi: 10.1186/s13046-019-1163-6 30999932 PMC6472102

[140] Song M He J Pan QZ Yang J Zhao J Zhang YJ. Cancer-associated fibroblast-mediated cellular crosstalk supports hepatocellular carcinoma progression. Hepatol (Baltimore Md) (2021) 73(5):1717–35. doi: 10.1002/hep.31792 33682185

[141] Zhao Z Bai S Wang R Xiong S Li Y Wang X. Cancer-associated fibroblasts endow stem-like qualities to liver cancer cells by modulating autophagy. Cancer Manage Res (2019) 11:5737–44. doi: 10.2147/cmar.S197634 PMC6598753 31296998

[142] Zhou Y Tang W Zhuo H Zhu D Rong D Sun J. Cancer-associated fibroblast exosomes promote chemoresistance to cisplatin in hepatocellular carcinoma through circzfr targeting signal transducers and activators of transcription (Stat3)/Nuclear factor -kappa b (Nf-Kb) pathway. Bioengineered (2022) 13(3):4786–97. doi: 10.1080/21655979.2022.2032972 PMC8973934 35139763

[143] Fang T Lv H Lv G Li T Wang C Han Q. Tumor-derived exosomal mir-1247-3p induces cancer-associated fibroblast activation to foster lung metastasis of liver cancer. Nat Commun (2018) 9(1):191. doi: 10.1038/s41467-017-02583-0 29335551 PMC5768693

[144] Lai SC Su YT Chi CC Kuo YC Lee KF Wu YC. Dnmt3b/Oct4 expression confers sorafenib resistance and poor prognosis of hepatocellular carcinoma through il-6/Stat3 regulation. J Exp Clin Cancer research: CR (2019) 38(1):474. doi: 10.1186/s13046-019-1442-2 31771617 PMC6878666

[145] Liu X Wang H Yang M Hou Y Chen Y Bie P. Exosomal mir-29b from cancer-associated fibroblasts inhibits the migration and invasion of hepatocellular carcinoma cells. Trans Cancer Res (2020) 9(4):2576–87. doi: 10.21037/tcr.2020.02.68 PMC8797999 35117617

[146] Jia W Liang S Cheng B Ling C. The role of cancer-associated fibroblasts in hepatocellular carcinoma and the value of traditional Chinese medicine treatment. Front Oncol (2021) 11:763519. doi: 10.3389/fonc.2021.763519 34868982 PMC8636329

[147] Romanque P Piguet AC Dufour JF. Targeting vessels to treat hepatocellular carcinoma. Clin Sci (London England: 1979) (2008) 114(7):467–77. doi: 10.1042/cs20070310 18302534

[148] Helmlinger G Yuan F Dellian M Jain RK. Interstitial ph and Po2 gradients in solid tumors in vivo: High-resolution measurements reveal a lack of correlation. Nat Med (1997) 3(2):177–82. doi: 10.1038/nm0297-177 9018236

[149] Xiong XX Qiu XY Hu DX Chen XQ. Advances in hypoxia-mediated mechanisms in hepatocellular carcinoma. Mol Pharmacol (2017) 92(3):246–55. doi: 10.1124/mol.116.107706 28242743

[150] Huang WJ Jeng YM Lai HS Fong IU Sheu FY Lai PL. Expression of hypoxic marker carbonic anhydrase ix predicts poor prognosis in resectable hepatocellular carcinoma. PloS One (2015) 10(3):e0119181. doi: 10.1371/journal.pone.0119181 25738958 PMC4349857

[151] Nardinocchi L Puca R Sacchi A D’Orazi G. Inhibition of hif-1alpha activity by homeodomain-interacting protein kinase-2 correlates with sensitization of chemoresistant cells to undergo apoptosis. Mol Cancer (2009) 8:1. doi: 10.1186/1476-4598-8-1 19128456 PMC2628864

[152] Sullivan R Paré GC Frederiksen LJ Semenza GL Graham CH. Hypoxia-induced resistance to anticancer drugs is associated with decreased senescence and requires hypoxia-inducible factor-1 activity. Mol Cancer Ther (2008) 7(7):1961–73. doi: 10.1158/1535-7163.Mct-08-0198 18645006

[153] Masoud GN Li W. Hif-1α pathway: Role, regulation and intervention for cancer therapy. Acta Pharm Sin B (2015) 5(5):378–89. doi: 10.1016/j.apsb.2015.05.007 PMC4629436 26579469

[154] Liu J Yang C Huang XM Lv PP Yang YK Zhao JN. Knockdown of fbi-1 inhibits the warburg effect and enhances the sensitivity of hepatocellular carcinoma cells to molecular targeted agents *Via* mir-3692/Hif-1α. Front Oncol (2021) 11:796839. doi: 10.3389/fonc.2021.796839 34869045 PMC8633402

[155] Gwak GY Yoon JH Kim KM Lee HS Chung JW Gores GJ. Hypoxia stimulates proliferation of human hepatoma cells through the induction of hexokinase ii expression. J Hepatol (2005) 42(3):358–64. doi: 10.1016/j.jhep.2004.11.020 15710218

[156] Amann T Maegdefrau U Hartmann A Agaimy A Marienhagen J Weiss TS. Glut1 expression is increased in hepatocellular carcinoma and promotes tumorigenesis. Am J Pathol (2009) 174(4):1544–52. doi: 10.2353/ajpath.2009.080596 PMC2671384 19286567

[157] Li S Li J Dai W Zhang Q Feng J Wu L. Genistein suppresses aerobic glycolysis and induces hepatocellular carcinoma cell death. Br J Cancer (2017) 117(10):1518–28. doi: 10.1038/bjc.2017.323 PMC5680469 28926527

[158] Bahar E Han SY Kim JY Yoon H. Chemotherapy resistance: Role of mitochondrial and autophagic components. Cancers (2022) 14(6):1462. doi: 10.3390/cancers14061462 35326612 PMC8945922

[159] Schönenberger MJ Kovacs WJ. Hypoxia signaling pathways: Modulators of oxygen-related organelles. Front Cell Dev Biol (2015) 3:42. doi: 10.3389/fcell.2015.00042 26258123 PMC4508581

[160] Sun L Li T Wei Q Zhang Y Jia X Wan Z. Upregulation of Bnip3 mediated by Erk/Hif-1α pathway induces autophagy and contributes to anoikis resistance of hepatocellular carcinoma cells. Future Oncol (London England) (2014) 10(8):1387–98. doi: 10.2217/fon.14.70 25052749

[161] Prieto-Domínguez N Méndez-Blanco C Carbajo-Pescador S Fondevila F García-Palomo A González-Gallego J. Melatonin enhances sorafenib actions in human hepatocarcinoma cells by inhibiting Mtorc1/P70s6k/Hif-1α and hypoxia-mediated mitophagy. Oncotarget (2017) 8(53):91402–14. doi: 10.18632/oncotarget.20592 PMC5710933 29207653

[162] Li S Yang Y Ding X Yang M She S Peng H. Lhbs can elevate the expression of Mdr1 through hif-1α in patients with chb infection: A comparative proteomic study. Oncotarget (2017) 8(3):4549–62. doi: 10.18632/oncotarget.13941 PMC5354853 27999186

[163] Tak E Lee S Lee J Rashid MA Kim YW Park JH. Human carbonyl reductase 1 upregulated by hypoxia renders resistance to apoptosis in hepatocellular carcinoma cells. J Hepatol (2011) 54(2):328–39. doi: 10.1016/j.jhep.2010.06.045 21056497

[164] Zhang Jian PY Ma Hai Yang Liu. Effect of Silymarin on Hif-1α and Expression in Hepg-2 Cells under Hypoxia. Electronic Journal of Metabolism and Nutrition (2021) 4:175-8.

[165] Lin Z Niu Y Wan A Chen D Liang H Chen X. Rna M(6) a methylation regulates sorafenib resistance in liver cancer through Foxo3-mediated autophagy. EMBO J (2020) 39(12):e103181. doi: 10.15252/embj.2019103181 32368828 PMC7298296

[166] Liang C Dong Z Cai X Shen J Xu Y Zhang M. Hypoxia induces sorafenib resistance mediated by autophagy *Via* activating Foxo3a in hepatocellular carcinoma. Cell Death Dis (2020) 11(11):1017. doi: 10.1038/s41419-020-03233-y 33250518 PMC7701149

[167] Che N Ng KY Wong TL Tong M Kau PW Chan LH. Prmt6 deficiency induces autophagy in hostile microenvironments of hepatocellular carcinoma tumors by regulating Bag5-associated Hsc70 stability. Cancer Lett (2021) 501:247–62. doi: 10.1016/j.canlet.2020.11.002 33186656

[168] Tang Y Zhang Y Liu S Sun Z Wang C Li L. 14-3-3ζ binds to and stabilizes phospho-beclin 1(S295) and induces autophagy in hepatocellular carcinoma cells. J Cell Mol Med (2020) 24(1):954–64. doi: 10.1111/jcmm.14806 PMC6933394 31709727

[169] Wu FQ Fang T Yu LX Lv GS Lv HW Liang D. Adrb2 signaling promotes hcc progression and sorafenib resistance by inhibiting autophagic degradation of Hif1α. J Hepatol (2016) 65(2):314–24. doi: 10.1016/j.jhep.2016.04.019 27154061

[170] Peng WX Xiong EM Ge L Wan YY Zhang CL Du FY. Egr-1 promotes hypoxia-induced autophagy to enhance chemo-resistance of hepatocellular carcinoma cells. Exp Cell Res (2016) 340(1):62–70. doi: 10.1016/j.yexcr.2015.12.006 26708617

[171] Wang Q Lu D Fan L Li Y Liu Y Yu H. Cox-2 induces apoptosis-resistance in hepatocellular carcinoma cells *Via* the hif-1α/Pkm2 pathway. Int J Mol Med (2019) 43(1):475–88. doi: 10.3892/ijmm.2018.3936 30365092

[172] Yan B Li T Shen L Zhou Z Liu X Wang X. Simultaneous knockdown of yap and taz increases apoptosis of hepatocellular carcinoma cells under hypoxic condition. Biochem Biophys Res Commun (2019) 515(2):275–81. doi: 10.1016/j.bbrc.2019.05.143 31146919

[173] Xia X Wang Q Ye T Liu Y Liu D Song S. Nrf2/Abcb1-mediated efflux and Parp1-mediated dampening of DNA damage contribute to doxorubicin resistance in chronic hypoxic Hepg2 cells. Fundam Clin Pharmacol (2020) 34(1):41–50. doi: 10.1111/fcp.12505 31420991

[174] Hashimoto N Tsunedomi R Yoshimura K Watanabe Y Hazama S Oka M. Cancer stem-like sphere cells induced from de-differentiated hepatocellular carcinoma-derived cell lines possess the resistance to anti-cancer drugs. BMC Cancer (2014) 14:722. doi: 10.1186/1471-2407-14-722 25260650 PMC4190290

[175] Pang Y Lin Y Wang X Wang J Liu Q Ding N. Inhibition of abnormally activated hif-1α-Glut1/3-Glycolysis pathway enhances the sensitivity of hepatocellular carcinoma to 5-caffeoylquinic acid and its derivatives. Eur J Pharmacol (2022) 920:174844. doi: 10.1016/j.ejphar.2022.174844 35189090

[176] Gao R Buechel D Kalathur RKR Morini MF Coto-Llerena M Ercan C. Usp29-mediated Hif1α stabilization is associated with sorafenib resistance of hepatocellular carcinoma cells by upregulating glycolysis. Oncogenesis (2021) 10(7):52. doi: 10.1038/s41389-021-00338-7 34272356 PMC8285469

[177] Ke X Chen Y Wang P Xing J Chen Z. Upregulation of Cd147 protects hepatocellular carcinoma cell from apoptosis through glycolytic switch *Via* hif-1 and mct-4 under hypoxia. Hepatol Int (2014) 8(3):405–14. doi: 10.1007/s12072-014-9536-6 26202642

[178] Song Y Zou X Zhang D Liu S Duan Z Liu L. Self-enforcing Hmgb1/Nf-Kb/Hif-1α feedback loop promotes cisplatin resistance in hepatocellular carcinoma cells. J Cancer (2020) 11(13):3893–902. doi: 10.7150/jca.42944 PMC7171489 32328193

[179] Liang Y Zheng T Song R Wang J Yin D Wang L. Hypoxia-mediated sorafenib resistance can be overcome by Ef24 through Von hippel-lindau tumor suppressor-dependent hif-1α inhibition in hepatocellular carcinoma. Hepatol (Baltimore Md) (2013) 57(5):1847–57. doi: 10.1002/hep.26224 23299930

[180] Zhu H Chen XP Luo SF Guan J Zhang WG Zhang BX. [the role of extracellular signal-regulated Kinase/Mitogen-activated protein kinase pathway in multidrug resistance of hepatocellular carcinoma]. Zhonghua wai ke za zhi [Chinese J surgery] (2007) 45(13):917–20. 17953842

[181] Zhang D Wu F Song J Meng M Fan X Lu C. A role for the Npm1/Ptpn14/Yap axis in mediating hypoxia-induced chemoresistance to sorafenib in hepatocellular carcinoma. Cancer Cell Int (2022) 22(1):65. doi: 10.1186/s12935-022-02479-0 35135548 PMC8822852

[182] Pouget JP Frelon S Ravanat JL Testard I Odin F Cadet J. Formation of modified DNA bases in cells exposed either to gamma radiation or to high-let particles. Radiat Res (2002) 157(5):589–95. doi: 10.1667/0033-7587(2002)157[0589:fomdbi]2.0.co;2 11966325

[183] Brown JM Wilson WR. Exploiting tumour hypoxia in cancer treatment. Nat Rev Cancer (2004) 4(6):437–47. doi: 10.1038/nrc1367 15170446

[184] Bai B Liu Y Fu XM Qin HY Li GK Wang HC. Dysregulation of Ezh2/Mir-138-5p axis contributes to radiosensitivity in hepatocellular carcinoma cell by downregulating hypoxia-inducible factor 1 alpha (Hif-1α). Oxid Med Cell Longevity (2022) 2022:7608712. doi: 10.1155/2022/7608712 PMC9444475 36071871

[185] Bamodu OA Chang HL Ong JR Lee WH Yeh CT Tsai JT. Elevated Pdk1 expression drives Pi3k/Akt/Mtor signaling promotes radiation-resistant and dedifferentiated phenotype of hepatocellular carcinoma. Cells (2020) 9(3):746. doi: 10.3390/cells9030746 32197467 PMC7140693

[186] Land SC Tee AR. Hypoxia-inducible factor 1alpha is regulated by the mammalian target of rapamycin (Mtor) *Via* an mtor signaling motif. J Biol Chem (2007) 282(28):20534–43. doi: 10.1074/jbc.M611782200 17502379

[187] Fang Y Zhan Y Xie Y Du S Chen Y Zeng Z. Integration of glucose and cardiolipin anabolism confers radiation resistance of hcc. Hepatol (Baltimore Md) (2022) 75(6):1386–401. doi: 10.1002/hep.32177 PMC9299851 34580888

[188] Bowyer C Lewis AL Lloyd AW Phillips GJ Macfarlane WM. Hypoxia as a target for drug combination therapy of liver cancer. Anti-cancer Drugs (2017) 28(7):771–80. doi: 10.1097/cad.0000000000000516 PMC5515631 28542038

[189] Gao C Wang S Shao W Zhang Y Lu L Jia H. Rapamycin enhances the anti-tumor activity of cabozantinib in cmet inhibitor-resistant hepatocellular carcinoma. Front Med (2021) 16:467–82. doi: 10.1007/s11684-021-0869-y 34669157

[190] Chang CC Dinh TK Lee YA Wang FN Sung YC Yu PL. Nanoparticle delivery of Mno(2) and antiangiogenic therapy to overcome hypoxia-driven tumor escape and suppress hepatocellular carcinoma. ACS Appl materials interfaces (2020) 12(40):44407–19. doi: 10.1021/acsami.0c08473 32865389

[191] Huang R Zhang L Jin J Zhou Y Zhang H Lv C. Bruceine d inhibits hif-1α-Mediated glucose metabolism in hepatocellular carcinoma by blocking Icat/B-catenin interaction. Acta Pharm Sin B (2021) 11(11):3481–92. doi: 10.1016/j.apsb.2021.05.009 PMC8642446 34900531

[192] Zhang J Peng Y Hai M. Effects of silymarin on HIF 1α and MDR1 expression in HepG 2 cells under hypoxia[J]. Electron J Metab Nutr Cancer (2021) 8:(2):175–8.

[193] Liu J Ren L Li S Li W Zheng X Yang Y. The biology, function, and applications of exosomes in cancer. Acta Pharm Sin B (2021) 11(9):2783–97. doi: 10.1016/j.apsb.2021.01.001 PMC8463268 34589397

[194] Fu X Liu M Qu S Ma J Zhang Y Shi T. Exosomal microrna-32-5p induces multidrug resistance in hepatocellular carcinoma *Via* the Pi3k/Akt pathway. J Exp Clin Cancer research: CR (2018) 37(1):52. doi: 10.1186/s13046-018-0677-7 29530052 PMC5846230

[195] Wei Y Lai X Yu S Chen S Ma Y Zhang Y. Exosomal mir-221/222 enhances tamoxifen resistance in recipient er-positive breast cancer cells. Breast Cancer Res Treat (2014) 147(2):423–31. doi: 10.1007/s10549-014-3037-0 25007959

[196] Wang M Wang Y Ye F Yu K Wei W Liu M. Exosome encapsulated ncrnas in the development of hcc: Potential circulatory biomarkers and clinical therapeutic targets. Am J Cancer Res (2021) 11(8):3794–812. PMC8414376 34522450

[197] Xue D Han J Liu Y Tuo H Peng Y. Current perspectives on exosomes in the diagnosis and treatment of hepatocellular carcinoma (Review). Cancer Biol Ther (2021) 22(4):279–90. doi: 10.1080/15384047.2021.1898728 PMC8183537 33847207

[198] Li W Xin X Li X Geng J Sun Y. Exosomes secreted by m2 macrophages promote cancer stemness of hepatocellular carcinoma via the mir-27a-3p/Txnip pathways. Int Immunopharmacol (2021) 101(Pt A):107585. doi: 10.1016/j.intimp.2021.107585 34601333

[199] Zhang PF Gao C Huang XY Lu JC Guo XJ Shi GM. Cancer cell-derived exosomal Circuhrf1 induces natural killer cell exhaustion and may cause resistance to anti-Pd1 therapy in hepatocellular carcinoma. Mol Cancer (2020) 19(1):110. doi: 10.1186/s12943-020-01222-5 32593303 PMC7320583

[200] Zhang K Shao CX Zhu JD Lv XL Tu CY Jiang C. Exosomes function as nanoparticles to transfer mir-199a-3p to reverse chemoresistance to cisplatin in hepatocellular carcinoma. Bioscience Rep (2020) 40(7):BSR20194026. doi: 10.1042/bsr20194026 PMC7341182 32463473

[201] Wang G Zhao W Wang H Qiu G Jiang Z Wei G. Exosomal mir-744 inhibits proliferation and sorafenib chemoresistance in hepatocellular carcinoma by targeting Pax2. Med Sci monitor: Int Med J Exp Clin Res (2019) 25:7209–17. doi: 10.12659/msm.919219 PMC6777417 31553714

[202] Turato C Fornari F Pollutri D Fassan M Quarta S Villano G. Mir-122 targets Serpinb3 and is involved in sorafenib resistance in hepatocellular carcinoma. J Clin Med (2019) 8(2):171. doi: 10.3390/jcm8020171 30717317 PMC6406326

[203] Sun Z Chen J Chen G Zhang C Li C. Recent advances of engineered and artificial drug delivery system towards solid tumor based on immune cells. Biomed materials (Bristol England) (2022) 17 :02202. doi: 10.1088/1748-605X/ac4c8b 35042206

[204] Zhang J Ji C Zhang H Shi H Mao F Qian H. Engineered neutrophil-derived exosome-like vesicles for targeted cancer therapy. Sci Adv (2022) 8(2):eabj8207. doi: 10.1126/sciadv.abj8207 35020437 PMC8754405

[205] Sayyed AA Gondaliya P Mali M Pawar A Bhat P Khairnar A. Mir-155 inhibitor-laden exosomes reverse resistance to cisplatin in a 3d tumor spheroid and xenograft model of oral cancer. Mol pharmaceutics (2021) 18(8):3010–25. doi: 10.1021/acs.molpharmaceut.1c00213 34176265

[206] Campos A Sharma S Obermair A Salomon C. Extracellular vesicle-associated mirnas and chemoresistance: A systematic review. Cancers (2021) 13(18):4608. doi: 10.3390/cancers13184608 34572835 PMC8472653

[207] Sato T Vries RG Snippert HJ van de Wetering M Barker N Stange DE. Single Lgr5 stem cells build crypt-villus structures in vitro without a mesenchymal niche. Nature (2009) 459(7244):262–5. doi: 10.1038/nature07935 19329995

[208] Pampaloni F Reynaud EG Stelzer EH. The third dimension bridges the gap between cell culture and live tissue. Nat Rev Mol Cell Biol (2007) 8(10):839–45. doi: 10.1038/nrm2236 17684528

[209] Blidisel A Marcovici I Coricovac D Hut F Dehelean C Cretu OJC. Experimental models of hepatocellular carcinoma-a preclinical perspective. Cancers (2021) 13(15):3651. doi: 10.3390/cancers13153651 34359553 PMC8344976

[210] Tharehalli U Svinarenko M Lechel A. Remodelling and improvements in organoid technology to study liver carcinogenesis in a dish. Stem Cells Int (2019) 2019:3831213. doi: 10.1155/2019/3831213 30915124 PMC6399527

[211] Huch M Dorrell C Boj SF van Es JH Li VS van de Wetering M. *In vitro* expansion of single Lgr5+ liver stem cells induced by wnt-driven regeneration. Nature (2013) 494(7436):247–50. doi: 10.1038/nature11826 PMC3634804 23354049

[212] Huch M Gehart H van Boxtel R Hamer K Blokzijl F Verstegen MM. Long-term culture of genome-stable bipotent stem cells from adult human liver. Cell (2015) 160(1-2):299–312. doi: 10.1016/j.cell.2014.11.050 25533785 PMC4313365

[213] Wang Y Takeishi K Li Z Cervantes-Alvarez E Collin de l’Hortet A Guzman-Lepe J. Microenvironment of a tumor-organoid system enhances hepatocellular carcinoma malignancy-related hallmarks. Organogenesis (2017) 13(3):83–94. doi: 10.1080/15476278.2017.1322243 28548903 PMC5654820

[214] Broutier L Mastrogiovanni G Verstegen MM Francies HE Gavarró LM Bradshaw CR. Human primary liver cancer-derived organoid cultures for disease modeling and drug screening. Nat Med (2017) 23(12):1424–35. doi: 10.1038/nm.4438 PMC5722201 29131160

[215] Nuciforo S Fofana I Matter MS Blumer T Calabrese D Boldanova T. Organoid models of human liver cancers derived from tumor needle biopsies. Cell Rep (2018) 24(5):1363–76. doi: 10.1016/j.celrep.2018.07.001 PMC6088153 30067989

[216] Sun L Wang Y Cen J Ma X Cui L Qiu Z. Modelling liver cancer initiation with organoids derived from directly reprogrammed human hepatocytes. Nat Cell Biol (2019) 21(8):1015–26. doi: 10.1038/s41556-019-0359-5 31332348

[217] Li L Knutsdottir H Hui K Weiss MJ He J Philosophe B. Human primary liver cancer organoids reveal intratumor and interpatient drug response heterogeneity. JCI Insight (2019) 4(2):e121490. doi: 10.1172/jci.insight.121490 30674722 PMC6413833

[218] Nuciforo S Heim MH. Organoids to model liver disease. JHEP reports: Innovation Hepatol (2021) 3(1):100198. doi: 10.1016/j.jhepr.2020.100198 PMC7672322 33241206

[219] Loh JJ Li TW Zhou L Wong TL Liu X Ma VWS. Fstl1 secreted by activated fibroblasts promotes hepatocellular carcinoma metastasis and stemness. Cancer Res (2021) 81(22):5692–705. doi: 10.1158/0008-5472.Can-20-4226 34551961

[220] Xian L Zhao P Chen X Wei Z Ji H Zhao J. Heterogeneity, inherent and acquired drug resistance in patient-derived organoid models of primary liver cancer. Cell Oncol (Dordrecht) (2022) 45 :1019–36. doi: 10.1007/s13402-022-00707-3 PMC12978112 36036881

[221] Dong H Li Z Bian S Song G Song W Zhang M. Culture of patient-derived multicellular clusters in suspended hydrogel capsules for pre-clinical personalized drug screening. Bioactive materials (2022) 18:164–77. doi: 10.1016/j.bioactmat.2022.03.020 PMC8961426 35387168

